# WoR^+^ Ontology: Modeling Data and Services in Web Connected Environments

**DOI:** 10.3390/s26030941

**Published:** 2026-02-01

**Authors:** Lara Kallab, Khouloud Salameh, Richard Chbeir

**Affiliations:** 1 XP3R Inc., Santa Ana, CA 92785, USA; 2 Computer Science and Engineering Department, American University of Ras Al Khaimah, P.O. Box 10021, Ras Al Khaimah 72603, United Arab Emirates; 3 Computer Science Department, Université de Pau et des Pays de l’Adour, E2S UPPA, LIUPPA, EA3000, 64600 Anglet, France

**Keywords:** Web connected environment, Web resource, service, data, semantic Web modeling, ontology, composition

## Abstract

The Web of Things (WoT) is a set of standards established by the World Wide Web Consortium (W3C) to enable interoperability across various Internet of Things (IoT) platforms. These standards facilitate seamless device-to-device interactions and application-to-application communication across heterogeneous environments. To identify and utilize resources, whether data or services, offered by Web-connected devices and applications, these resources must be described using an open, shared, and dynamic knowledge representation capable of supporting both syntactic and semantic interoperability. In this paper, we present WoR^+^, a Web of Resources ontology based on a modular and unified vocabulary for describing Web resources (Web services and Web data). WoR^+^ offers several advantages: (a) it supports the discovery, selection, and composition of data and services provided by Web-connected devices and applications; (b) it provides reasoning capabilities for inferring new knowledge; and (c) it supports extensibility and adaptability to emerging domain requirements. Experimental evaluation shows that WoR^+^ ontology achieves high effectiveness, strong performance, and good clarity and consistency.

## 1. Introduction

The Internet of Things (IoT) refers to physical objects, such as smart devices, equipped with sensors, software, and communication technologies that enable them to collect and exchange data over the Internet [1]. Due to the heterogeneity of these objects, it remains difficult to establish seamless communication to enable proper interoperability. Similar challenges arise when applications are developed on different platforms and in different programming languages. To address these challenges, the Web [2] serves as a unifying communication layer that enables interaction among diverse connected objects and applications. As a result, the Web has now become a key communication platform [3], which has led to the development of the Web of Things [4] (WoT).

Within the Web ecosystem, REST (Representational State Transfer) [5] has been defined as an architectural style that standardizes interactions among Web components, allowing simpler, more consistent, and efficient access to Web resources. In REST, data and services provided by objects and applications are exposed as resources. A resource is a fundamental building block that represents a specific item of data (e.g., text, images, videos) or a Web service (e.g., temperature sensor Web service, smart light control Web service). Some of the key benefits of using resources in REST include the following:

- Scalability: Resources are designed as self-contained entities that can be accessed and manipulated independently. This makes it easier to scale the environment to handle large numbers of requests and to add or remove resources as needed.
- Ease of identification: Resources are identified by URIs (Uniform Resource Identifiers), providing a global addressing space for resource discovery.
- Flexibility: Resources can be easily extended to support new features and capabilities, making it easier to evolve the Web environment over time.

However, REST alone does not fully address the interoperability challenges. Resources must be properly defined and represented to ensure widespread use. This is where Semantic Web technologies come into play. Semantic Web technologies provide a common framework that enables machines to interpret and share domain knowledge, facilitating more effective information sharing and integration between different systems and platforms [6]. This is achieved by expressing and exchanging knowledge using clear and machine-understandable vocabularies that ensure consistent meaning across systems. One of the foundational components of Semantic Web technologies is ontologies [7], which provide a common and understandable vocabulary through a formal, explicit specification of a domain’s conceptualization. Ontologies facilitate knowledge transfer between different systems and organizations, offering both syntactic and semantic interoperability.

To describe IoT-/WoT-based domains, including their services and data, numerous models, particularly ontology-based ones [8,9,10,11], have been proposed over the past decade. These models serve various purposes, such as representing specific domains of knowledge (e.g., entities, relations, sensor data, or device capabilities) in a machine-understandable way, and supporting the discovery, aggregation, and remote accessibility of things, comprising services and data. However, these models have several limitations. Many are primarily services-oriented [9,11,12,13], and do not describe the data that services consume or produce. Others overlook application-level services or fail to capture important semantic links between services, which are essential for composition and substitution.

In addition, most service-oriented models [14,15], include the necessary concepts to represent a service provided by an object (e.g., their locations, provided functions with required inputs and outputs, etc.); however, they fail to account for other essential aspects related to service links (e.g., a link indicating that one service is similar to another because it provides the same function). These links can facilitate the composition of services to create new value-added services when no single service can meet certain user demands. Furthermore, most service-oriented models omit the definition of composed services, which could be valuable in various use cases. Although some models provide descriptions of composite services, these are generally limited and do not follow the principles of the REST architecture [16]. This is significant, as many current models use SOAP-based Web services [17], which are heavier for IoT/WoT devices [18] compared to REST-based services.

As for data-oriented models  (https://www.talend.com/products/data-fabric/ (accessed on 28 January 2026) and https://www.dublincore.org/ (accessed on 28 January 2026)) [19,20,21], they primarily focus on representing data only and exclude the description of the services or sources that provide them. Moreover, despite covering numerous data specifications, including data links, they lack consideration of other important features, such as data composition, which enables the creation of new value-added information that did not originally exist, and data quality aspects (e.g., accuracy and reliability) to help in the selection of the best data when multiple data sources relate to the same subject of interest.

To overcome these limitations, this article introduces WoR^+^ (short for Web of Resources), an ontology-based Web resource model to describe both services and data exposed by devices and Web applications. It is designed to: (1) store and integrate resource specifications for various objects and applications; (2) simplify the process for users to discover, select, and compose exposed resources; (3) offer reasoning capabilities to uncover new information; and (4) support future adaptation and extensibility.

The remainder of the paper is organized as follows. Section 2 presents the motivation for the relevance and usability of our research. Section 3 reviews related work and highlights the originality of our contribution. Section 4 details our proposed Web resource model ontology. Section 5 evaluates the effectiveness, performance, clarity, and consistency of the proposed solution. Section 6 concludes the paper and provides future directions.

## 2. Motivating Scenario

**Figure 1:**
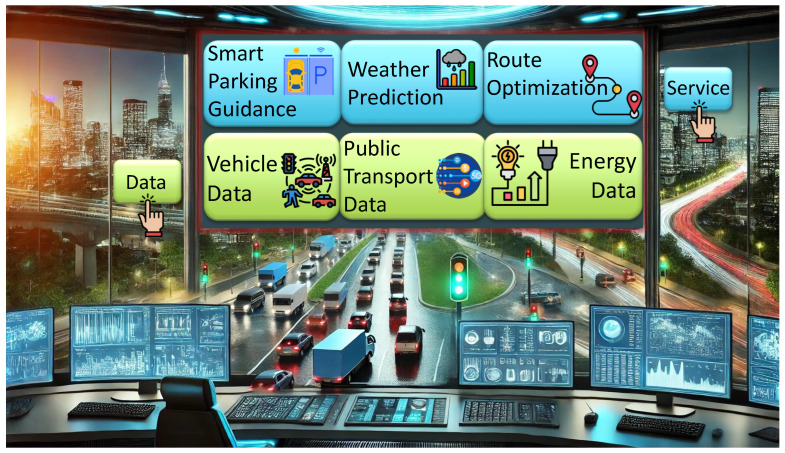
Examples of resources: data and services, provided by the Smart Traffic Management System (STMS).

To highlight the importance of our work, we present a scenario in which a Smart Traffic Management System (STMS) is deployed in a smart city to enhance urban mobility, reduce congestion, and optimize traffic flow. This system integrates various resources, including data resources (e.g., GPS data from vehicles such as private cars, taxis, and buses, as well as public transportation schedules) and service resources (e.g., weather forecasting, route optimization, and smart parking guidance), as illustrated in Figure 1.

In our scenario, we consider the city traffic manager to be responsible for monitoring and improving daily traffic conditions using interconnected data sources and intelligent services. To prevent congestion, particularly during peak hours, the traffic manager relies on three primary categories of data sources that can be selected from the STMS:

(a) Real-time traffic data, including GPS trajectories, vehicle speed, and flow information collected from public transportation systems, ride-sharing fleets, and private vehicles.
(b) Real-time weather data, which capture environmental parameters such as precipitation levels, visibility, temperature, and wind speed, are known to influence driving behavior and traffic conditions.
(c) Historical datasets, comprising long-term traffic records, recurrent congestion patterns, known bottlenecks, and the impact of past weather-related events on traffic flow.

**Figure 2:**
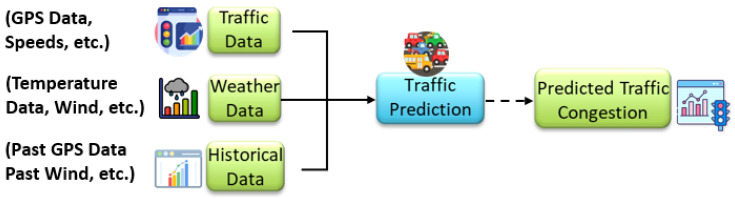
Data and services composition for traffic congestion prediction.

These heterogeneous datasets are ingested by the Traffic Prediction Service, which applies advanced machine learning models to estimate future traffic congestion levels and identify potential traffic jams in specific city zones (see Figure 2). The output is typically a congestion heatmap, annotated with timestamps and geospatial metadata, reflecting the predicted severity of congestion across different road segments. Figure 2 shows how the selected data sources and service are interconnected. This predictive output is then treated as a standalone data point that can be consumed by other smart city services. For example, the Route Optimization Service leverages congestion forecasts to dynamically compute and suggest alternative routes for various user profiles, including emergency response teams, public transit operators, and individual commuters. By integrating predicted congestion levels with road conditions and travel priorities, the system enhances urban mobility, reduces travel time, and contributes to more efficient and responsive traffic management.

However, despite the advantages of integrating diverse resources (both data and services), selecting and composing them remains complex due to factors such as diversity, redundancy, and limitations in users’ knowledge. These challenges can be described as follows:

- **Challenge A: Service Functionality Overlap.** Multiple traffic prediction services may offer similar core functionalities, such as estimating congestion levels or forecasting traffic flow, but often differ in the types of input data they consume (e.g., GPS trajectories, sensor networks, or real-time weather feeds) and the algorithms they employ (e.g., statistical models, deep learning, or hybrid approaches). This functional overlap complicates the selection process, making it difficult to identify the most suitable service for a given use case, particularly when user requirements vary in accuracy, latency, spatial resolution, or contextual awareness.
- **Challenge B: Data Redundancy and Selection Complexity.** Multiple datasets often exist in different structures and formats, yet some contain identical or highly similar information, complicating the selection process. For example, traffic congestion data may come from various sensors, such as cameras, radar, and loop detectors, that monitor the same intersections. Since each sensor provides information on traffic volume and vehicle speed, it is essential to identify differences between datasets in terms of accuracy and response time.
- **Challenge C: Overlap Between Data and Services.** In many cases, datasets and services provide similar information. For example, both traffic sensor data (e.g., loop detectors or camera data) and predictive analytics services (which use historical traffic data to forecast congestion) offer information about traffic conditions. While traffic sensors provide real-time insights, predictive analytics services generate forecasts based on historical trends. Selecting the best resource for a traffic management system requires considering factors such as: (i) Cost: Real-time sensor data may be more expensive to collect, (ii) Quality: Predictive analytics may offer more accurate forecasts but may be less reliable for real-time decisions, and (iii) Computational Efficiency: Processing real-time sensor data may require more computational resources compared to using pre-processed predictive data.
- **Challenge D: User Knowledge Limitations.** Traffic operators and city planners often lack in-depth technical knowledge regarding the functionalities, capabilities, and trade-offs of available services and datasets. This knowledge gap can hinder informed decision-making when selecting and integrating resources effectively. For example, various services can optimize traffic signal timing using inputs such as real-time traffic flow, historical traffic data, or weather forecasts. However, traffic operators may struggle to evaluate each service’s performance under different conditions (e.g., rush-hour congestion or sudden weather changes) or to understand technical specifications (e.g., processing speed, accuracy, and data requirements). This lack of specialized knowledge can lead to suboptimal resource selection, in which traffic management decisions are based on incomplete or less effective data and services.

To address these challenges, we propose a unified description model that is both human- and machine-readable, designed to accurately represent available resources (both data and services). Our model facilitates resource discovery and selection by: (i) ensuring interoperability between heterogeneous datasets and services, (ii) reducing ambiguity in resource descriptions to enable seamless integration, and (iii) providing a structured and comprehensible representation to support automated reasoning and decision-making. By offering a standardized framework for describing resources, this model enhances the efficiency of any environment that provides data and/or services, such as the STMS. The proposed model should meet the following criteria:

1. **A Thorough Model**. Given the variety of data and services within a connected Web environment like the STMS, the model must accommodate their descriptions to meet diverse user demands. Specifically:
  - **Service and Data-Oriented:** The STMS integrates various services to optimize traffic flow, such as real-time congestion monitoring, accident detection, and adaptive traffic light control. Understanding the functions and specifications of these services is crucial for traffic operators to select the most suitable ones. Additionally, some traffic-related data (e.g., vehicle counts from roadside sensors) may not be encapsulated or returned by any service. For instance, a smart sensor at an intersection may collect traffic density data and store it in a CSV file. To access this data and determine if it meets traffic control requirements, a formal description of the data is necessary to facilitate its discovery and selection.
  - **Object and Application Resources:** Since the STMS relies on a mix of objects like physical sensors (e.g., cameras, radar sensors) and Web applications (e.g., weather forecasting services, navigation apps), the model must describe resources provided by these diverse sources. Common concepts should be used to describe all types of resources, regardless of their origin. For example, “Timestamp” metadata can be associated with any type of resource (data or service), regardless of whether it is exposed as an IoT sensor or a weather application. Other attributes, however, may be specific to certain types of resource-based on their origins. For instance, “Location” and “Operation Range” would apply specifically to resources provided by objects.
  - **Composed Resources:** Some user demands in traffic management cannot be met by a single resource but require a combination of multiple resources (see composition example in Figure 2). To ensure the reusability of composite resources and avoid unnecessary composition costs (time, CPU, memory, etc.) when building them from scratch, storing and representing them within the model allows them to be treated as a single resource for future use.
  - **Virtual Resources:** Some traffic scenarios require services or data that are not directly available from physical devices or existing applications. For example, if a city lacks certain sensors at key intersections due to budget constraints, virtual resources can simulate traffic flow based on historical data and predictive modeling. Describing such virtual resources within the model enhances system capabilities when real-world data are incomplete.
  - **Resource Category:** To facilitate efficient discovery and selection of resources, data, and services in the STMS, they should be categorized. For instance, when collecting data, the traffic control system can more easily access resources in the “Data Collection” category to explore the best options. Categorization helps optimize resource management by enabling effective filtering, improving usability, and simplifying updates across related resources.
2. **An Expressive Model**. Each resource should be described with precision to ensure its appropriate use across various contexts. Below are key elements for describing services, data, and general resources (applicable to both services and data):
  - **Service Elements:**
    - **Provided Function:** The primary attribute for a service, indicating its function. For instance, a service for predicting traffic congestion must be explicitly labeled so operators can identify it and reroute traffic.
    - **I/O:** Services should specify the type of data they accept and produce. For example, a service that converts GPS-based vehicle location data into traffic-density metrics must define its input format (e.g., raw GPS logs) and output format (e.g., a heatmap of congestion levels). Clearly defined service I/O descriptions ensure efficient interoperability between services. For instance, an I/O type mismatch, such as one service using float data while another expects time-series data, can affect the accuracy of the predictions.
    - **Service Actions:** In certain cases, services can act on other resources, such as data or services, to achieve specific outcomes. For example, a “Traffic Light Adjustment” service may adjust light timings based on congestion data from a prediction service. These actions enable dynamic interactions between services, ensuring adaptive behavior and efficient resource management in the system.
    - **Service Links:** Services in the STMS often depend on one another. For instance, an “Emergency Vehicle Detection” service relies on real-time traffic camera feeds, while an “Adaptive Traffic Signal Control” service depends on congestion prediction services. Defining these dependencies through service links ensures seamless integration and makes service replacement easier when needed.
  - **Data Elements:**
    - **Data Content:** This refers to the information contained in a data resource. In the context of an STMS, data content may include sensor readings (e.g., temperature, humidity, vehicle count, speed) as well as contextual information (e.g., traffic congestion levels, incident severity). A well-structured representation of data content is essential for enabling efficient integration, querying, and reasoning across heterogeneous sources.
    - **Data Links:** Similar to services, data elements in the STMS may be interrelated. For example, vehicle speed data collected from road sensors may complement real-time accident reports. Establishing links between such datasets enhances data discovery and utilization.
  - **Resource Elements:**
    - **Location:** Given that traffic conditions vary by region, the model should account for the geographic context of data and services. For instance, traffic sensor data from downtown areas may be more relevant for congestion management than suburban traffic flow data when optimizing city-center navigation.
3. **A Model Supporting Resource Quality Aspects**. When multiple services or datasets provide similar functionality, selecting the most suitable one is crucial. For example, various road sensors may measure vehicle speed, and multiple sources may report accident incidents. The model should define Quality of Resource (QoR) attributes to aid in selecting the best resources; some are related to services (Quality of Service), and others are related to data (Quality of Data):
  - **Quality of Service:**
    - **Physical Level:** It refers to the characteristics of the physical objects offering the service (e.g., operational range and battery life). Such characteristics can be very useful in determining which resource is better to use. For example, choosing sensors with optimal accuracy is vital for reliable traffic monitoring.
    - **Network Level:** It represents the quality of data exchanged between the services of objects or applications. For example, the bandwidth and latency attributes of data transmission are crucial, especially for real-time applications like emergency response routing. Low-latency communication ensures timely traffic updates.
    - **Application Level:** It describes the quality of services provided by an object or an application, such as usage and response time. For example, services frequently used by traffic operators may indicate higher reliability. For instance, an AI-based congestion prediction service with a high usage rate may be preferred over less frequently used alternatives.
  - **Quality of Data:** Similar to service quality, it is important to define quality attributes associated with data. For example:
    - **Accuracy:** This ensures that the data is precise (e.g., avoiding misclassified vehicle types in automatic detection systems).
    - **Reliability**: This confirms that the data originates from trusted sources, such as government traffic control centers, rather than crowd-sourced applications.
    - **Timeliness:** This ensures that the data is up to date. For instance, an up-to-date real-time traffic feed is particularly critical for congestion management and accident response.

In addition to all the aforementioned criteria, most connected Web environments, including STMS, face challenges related to shared storage and resource access bottlenecks. For instance, as the number of connected traffic objects (e.g., sensors) and applications increases, centralized storage struggles to meet the growing demand, leading to performance degradation. Furthermore, multiple services may need to access the same data simultaneously, resulting in race conditions that can lead to data inconsistencies. By decoupling data and services, our proposed resource description model allows for independent scaling of resources. The ontology model also facilitates concurrency control by applying specific mechanisms. For example, a versioning system ensures that each data update creates a new version, enabling services to operate on distinct versions without conflicts. This approach prevents inconsistencies caused by concurrent modifications, thereby ensuring system reliability.

In light of the challenges and criteria outlined above, we introduce in this paper a semantic Web resources model (ontology), referred to as WoR^+^, which is detailed in Section 4. The ontology is structured using a vocabulary that is both human-readable and machine-readable, facilitating resource identification, selection, and composition.

## 3. State of the Art

In this section, we examine several IoT/WoT-based models, as well as data description-oriented models originally designed to ensure interoperability between solutions from different providers and across various sectors. These models semantically describe services and/or data, thereby enabling systems to be aware of their environment, track its evolution, and understand the changes they can effect. While discussing their primary core concepts and relationships, we compare these models using the criteria presented in Section 2. These criteria, categorized into three groups as shown below, mainly pertain to the concepts and properties used to model the services and data provided by “Things” (such as Web objects and Web applications):

1. **Thorough model:** It expresses the model’s capability to describe various types of resources:
  - Service and data-oriented, indicating the ability to represent resources that can be either services or data.
  - Objects and application resources, referring to resources (i.e., services and data) that can be provided by connected objects or connected applications.
  - Elementary resources, referring to resources (services or data) that are independent of other resources and whose behavior is not simulated.
  - Complex types of resources, i.e., composed resources and virtual resources.
  - Categories, referring to resource categories (e.g., “Data Collection Services” and “Data Collection”), which can facilitate the exploration of the resources provided by a Web environment and enhance the understanding of their behavior.
2. **Expressiveness:** demonstrating the model’s ability to encompass diverse criteria that represent a resource (service or data):
  - **A resource as a service**:
    - Function, denoting the task performed by the service.
    - I/O, designating the inputs and outputs of the service.
    - Actions, representing the effects that a service may produce upon execution.
    - Service links, referring to the links between services offered by connected objects and applications.
  - **A resource as a data**:
    - Data content, denoting the information contained within a data resource.
    - Data links, referring to the links between data provided by connected objects and applications.
  - **A resource as a service or a data**:
    - Resource location, designating the location of the object (providing services or data).
3. **Resource quality:** It indicates the model’s capability to specify the qualities of resources (services and data):
  - Quality of service, designating the quality attributes relative to a service. For example, physical quality aspects, such as battery life and operational range, or network quality aspects, such as bandwidth and latency.
  - Quality of data, referring to the quality attributes relative to data, e.g., accuracy and reliability.

### 3.1. IoT/WoT-Based Models

**Figure 3:**
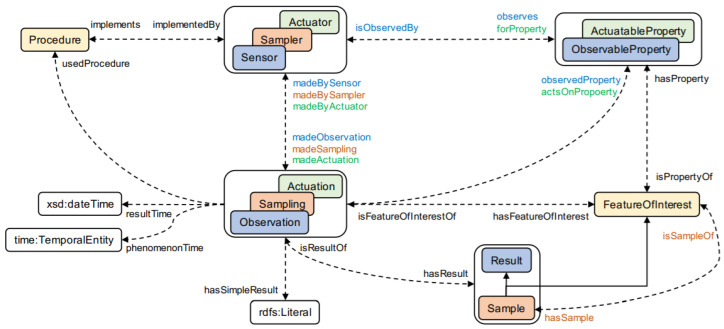
Overview of the SOSA/SSN ontology.

Released as W3C recommendations and OGC (Open Geospatial Consortium) implementation standards, SSN (Semantic Sensor Network) [22] and SOSA (Sensor, Observation, Sample, and Actuator) [23] are ontologies that describe sensors, actuators, and samplers, along with their associated observations, actions, and sampling processes. These ontologies feature a modular design, with SOSA serving as a self-contained core that is expanded by SSN and other modules to enhance expressivity and coverage. SSN and SOSA focus primarily on the physical elements of the IoT ecosystem (such as sensors, actuators, and samplers) and the modeling of related results, as depicted in Figure 3. HSSN [24] is an ontology that builds on SSN by incorporating concepts and properties related to sensor mobility and multimedia data. It addresses (i) sensor diversity, (ii) platform diversity, and (iii) data diversity. However, the definitions of interfaces, services (or operations), and data for the described IoT devices are not covered in any of these three models.

**Figure 4:**
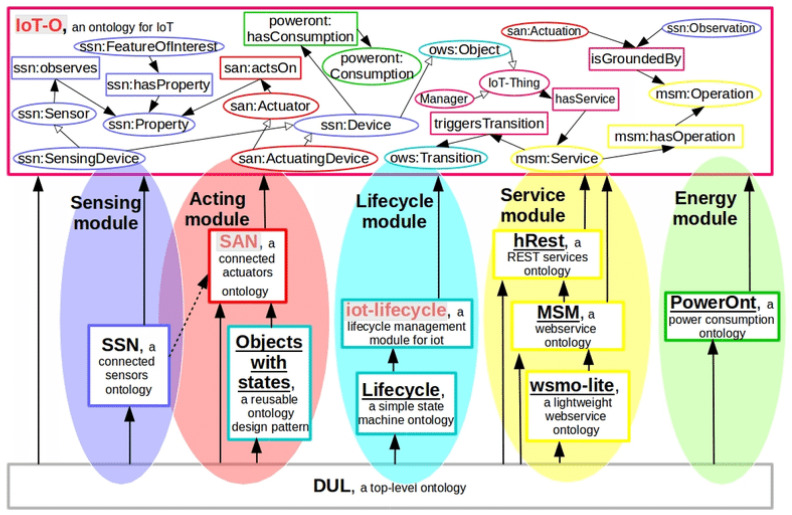
IoT-O main modules and concepts.

IoT-O [8] is a core-domain modular ontology designed for IoT, offering a vocabulary to describe connected devices and their interactions with their environment. As depicted in Figure 4, it consists of five modules: (1) the Sensing module, which leverages the SSN ontology [22] to detail sensors and their observations; (2) the Acting module, based on the SAN ontology  (https://www.irit.fr/recherches/MELODI/ontologies/SAN.html, accessed on 28 January 2026), which outlines how IoT devices interact with the physical world through their actions; (3) the Lifecycle module, which uses state machines to define the lifecycle and usage of IoT devices; (4) the Service module, which employs MSM (https://lov.linkeddata.es/dataset/lov/vocabs/msm, accessed on 28 January 2026), a REST-based ontology [16], to describe the services offered by IoT devices; and (5) the Energy module, defined by PowerOnt [25], which represents power consumption profiles for appliances. DUL (http://www.ontologydesignpatterns.org/ont/dul/DUL.owl, accessed on 28 January 2026) (DOLCE and DnS Ultralite) serves as an upper ontology for these five modules. In the IoT-O model, any connected entity, whether a physical object (ssn: Device) or an application (iot-o: Manager), can offer a Service. Each Service is assigned an address and can perform Operations accessible via HTTP methods (e.g., GET and PUT). These operations may include inputs, outputs, and hyperlinks to connect outputs to other operations. Despite that, IoT-O effectively describes REST services exposed by connected devices and applications, but it does not fully address composed or virtual services, their interconnections, or QoS aspects at the Network and Application levels. Moreover, it is not a data-oriented ontology.

**Figure 5:**
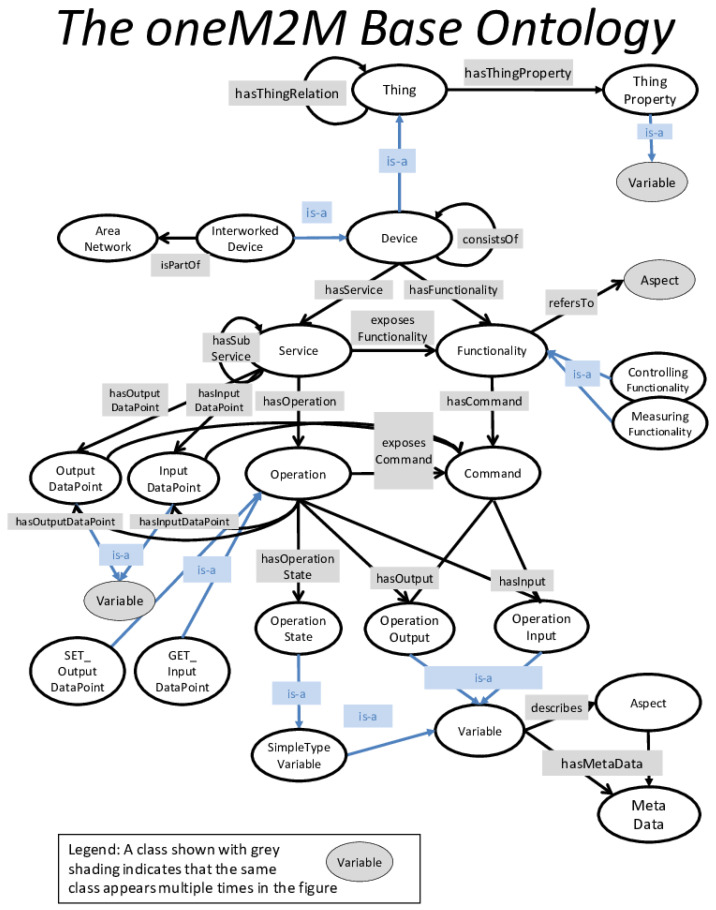
oneM2M Base ontology.

oneM2M (https://www.onem2m.org/technical/onem2m-ontologies, accessed on 28 January 2026) is a global standard for Machine-to-Machine (M2M) communications and IoT, developed through an open and collaborative process by various companies. It enables the annotation of application-specific resources (M2M data) with semantic descriptions. The standard defines a Base top-level ontology, as depicted in Figure 5, which facilitates the creation of subclasses or equivalence classes for application-level ontologies, such as the Smart Appliances REFerence Ontology (SAREF) (https://saref.etsi.org/core/v3.1.1/, accessed on 28 January 2026). In the oneM2M Base ontology, a Device offers a Service that provides Operations. Each Operation includes Operation Inputs and Outputs, represented as Variables that describe specific Aspects, such as Temperature. oneM2M is primarily focused on machine-to-machine interactions, without covering services exposed by Web applications, and resource (services or data) composition, virtualization, and quality aspects.

**Figure 6:**
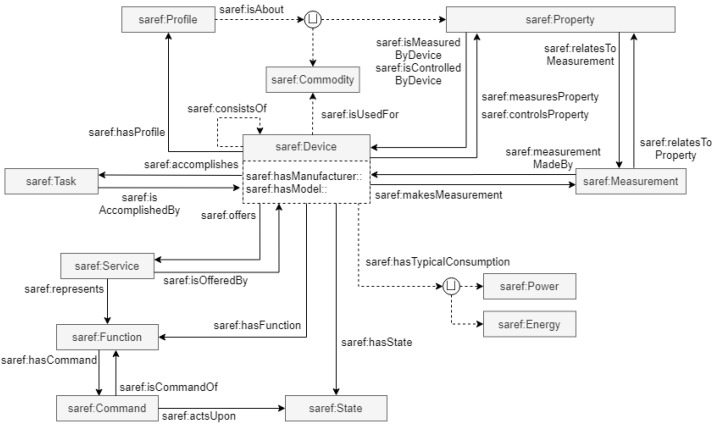
Overview of the SAREF ontology.

SAREF (Smart Appliances REFerence Ontology) (https://saref.etsi.org/, accessed on 28 January 2026) is a modular, domain-agnostic semantic framework designed for smart appliances. It defines key concepts (e.g., Device, Function, Command, Service, Measurement, Property, and Unit of Measure), their interrelationships, and axioms that govern their use. An overview of the SAREF ontology is provided in Figure 6. In SAREF, a Service is associated with one or more Functions and is provided by a Device that aims to make its functions discoverable, registerable, and remotely controllable by other devices on the network. While SAREF focuses primarily on the functions and measurements associated with devices, it does not address application services, service composition, virtualization, or QoS aspects. In addition, it is not a data-oriented ontology model.

WoT TD (Thing Description) [14] is a formal model and standard representation for the Web of Things developed by the W3C’s WoT working group. It outlines the metadata and interfaces of Things, which are abstractions of physical or virtual entities that interact within the Web of Things. Thing Descriptions use a streamlined vocabulary to define interactions, enabling the integration of various devices and facilitating interoperability among different applications. These descriptions can be encoded in JSON or JSON-LD formats, providing a machine-readable representation of information about the things.

**Figure 7:**
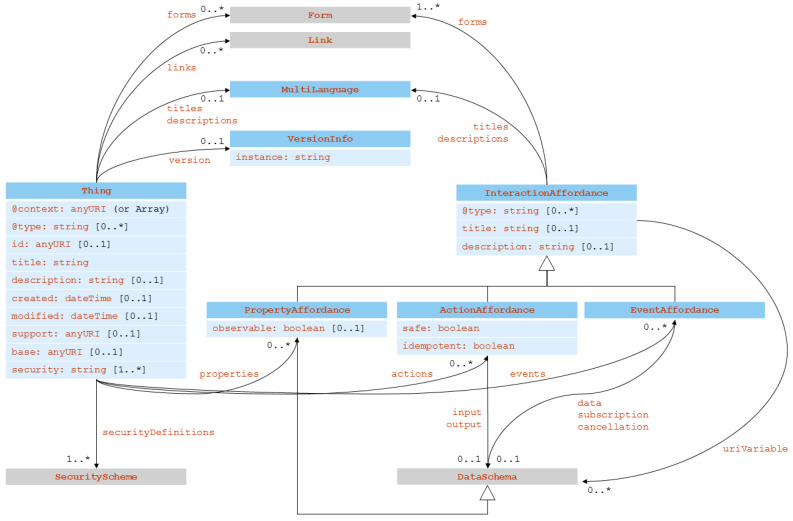
WoT TD core vocabulary.

The WoT TD (Thing Description) model relies on four key vocabularies: (1) the core TD Vocabulary, which captures the WoT concepts of Properties, Actions, and Events; (2) the Data Schema Vocabulary, which incorporates JSON Schema terms for defining data types and validation; (3) the WoT Security Vocabulary, which outlines security mechanisms and configuration requirements; and (4) the Hypermedia Controls Vocabulary, which describes RESTful communication principles through Web links and forms. Figure 7 provides an overview of the core vocabulary of WoT TD. In this model, Action denotes the functionality of a Thing, Property pertains to the sensing and control of parameters, Form indicates how to access a function, and Links connect things based on Web link specifications. Apart from not being a data-oriented model, and while it supports the description of objects and application services, the WoT TD model does not address device location, service I/O, service composition or virtualization, potential service links, or service quality.

**Figure 8:**
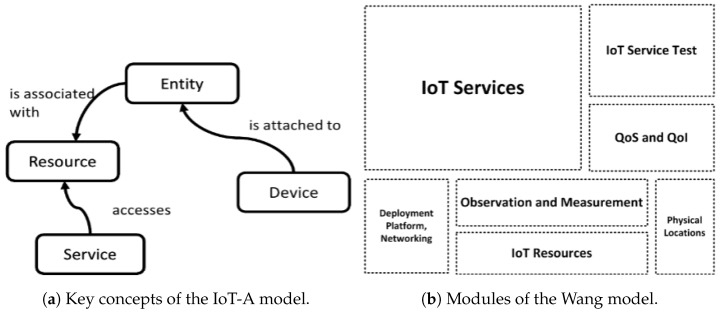
IoT-A and Wang ontology models.

The IoT-A model [12], illustrated in Figure 8a, defines key concepts within the IoT domain and their interrelationships. The primary concepts include Entity, Device, Resource, and Service. An Entity represents any object in the Internet of Things, such as a human, animal, or vehicle. A Device facilitates the entity’s integration into the digital world by enabling interactions. A Resource is a software component that either provides information about the Entity or controls the Device. A Service offers a standardized interface with well-defined functionalities for interacting with entities. While the IoT-A model establishes a foundational architecture for IoT projects, it does not address aspects such as application services, service composition and virtualization, potential service links, or quality of service considerations. Additionally, IoT-A is not a data-oriented model.

The Wang model [9] is an ontology designed to semantically describe knowledge within the IoT domain. As illustrated in Figure 8b, it consists of seven modules: (1) the IoT Services module, which details the functionalities of resources exposed by the devices; (2) the IoT Resources module, which builds upon the SSN ontology by incorporating additional important resources such as actuators; (3) the Observations and Measurements module, which describes the real data generated by IoT systems; (4) the Physical Locations module, which aids in locating and discovering IoT devices; (5) the Deployment Platform Networking module, which describes the organization, deployment, and system formation of IoT resources; (6) the Quality of Services and Quality of Information module, which includes concepts crucial for service composition and adaptation for IoT service providers and consumers; and (7) the IoT Service Test module, which supports the testing and verification of both functional and non-functional capabilities of IoT services during their design and deployment. However, the Wang model does not address application-specific services, composed or virtual services, or their potential links. Moreover, it is not a data-oriented model that allows linking data together and creating/storing new value-added data.

**Figure 9:**
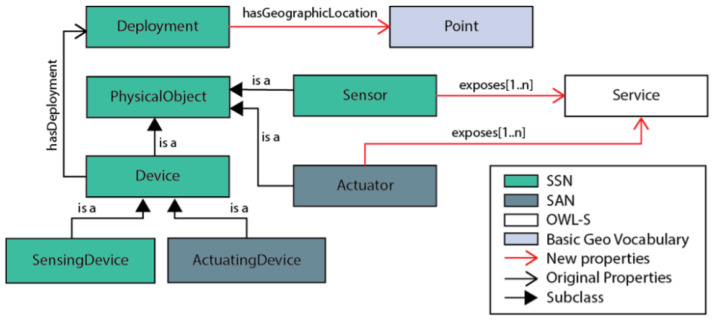
Overview of the ForwardDS-IoT model.

ForwardDS-IoT [11] is a semantic description model that leverages existing ontologies to represent IoT objects. It uses the SSN and SAN ontologies for object descriptions, the “Basic Geo” vocabulary (https://www.w3.org/2005/Incubator/geo/XGR-geo/, accessed on 28 January 2026) for geographical information such as latitude, longitude, and altitude, and the OWL-S ontology (https://www.w3.org/Submission/OWL-S/, accessed on 28 January 2026) for detailing IoT services. An overview of the ForwardDS-IoT model is shown in Figure 9. However, this model does not address the description of application services, virtual services, or their potential links. Additionally, ForwardDS-IoT is not a data-oriented model.

**Figure 10:**
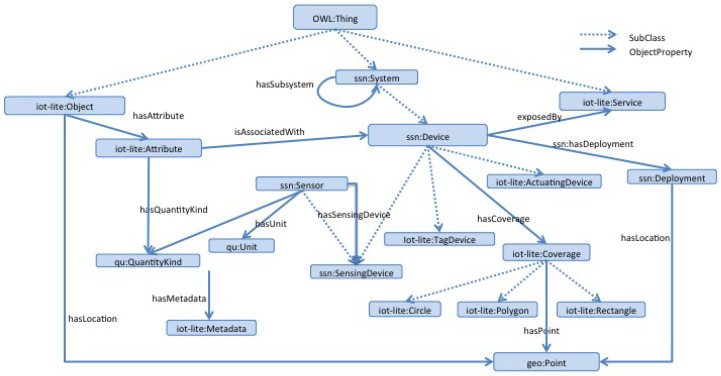
IoT-Lite ontology.

The SSN ontology, while comprehensive, may be considered “too complex” for dynamic environments due to its extensive number of concepts, many of which might not be utilized. To address this, IoT-Lite [26] simplifies the SSN ontology, focusing on essential IoT concepts to facilitate interoperability and sensory data discovery across diverse IoT platforms through a more streamlined semantic approach. Figure 10 depicts the core concepts of the IoT-Lite model, organized into three main classes: Object, System, and Service, with the latter being only briefly detailed. Similar to SSN, and besides being a non-data-oriented model, IoT-Lite emphasizes the physical aspects of the IoT domain but does not address the definitions of IoT devices’ services or operations.

**Figure 11:**
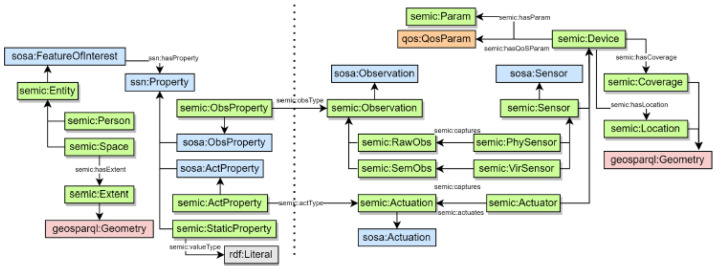
The Semic ontology supporting the description of a smart space.

A semantic ontology model called “Semic” is introduced in [13] to enhance interoperability in IoT environments. The Semic ontology acts as a mediator, providing a shared understanding of data, devices, and services within smart spaces. It captures the relationships between IoT components, enabling context-aware reasoning and automated decision-making. Semic outlines the core components of any smart space, such as entities (e.g., people and spaces), devices, observations, and actuations. It extends the SSN/SOSA ontology, enabling the automatic translation of user-defined actions based on higher-level concepts into corresponding device-level actions. An overview of this ontology model is given in Figure 11. However, the Semic model fails to account for application-specific services, including composed services, and their possible semantic links. Additionally, it is not a data-centric model that facilitates linking the devices’ shared data or semantically combining them to generate and store new value-added data.

The research in [27] proposes a semantic and ontology-based framework to enhance interoperability and automation in Internet of Things (IoT) systems. The framework aims to (i) enable efficient data exchange among heterogeneous IoT devices, (ii) improve communication speed (by 50%) and decision-making accuracy (by 12%), and (iii) support scalability across large IoT networks. The approach leverages semantic technologies and ontologies to address integration challenges among diverse IoT devices and to facilitate automated processes. The work primarily focuses on semantic modeling for device interoperability and data exchange, rather than explicitly describing: (1) services exposed by IoT devices (e.g., operations or functions they offer), (2) the data they provide (e.g., sensor measurements with associated metadata), or (3) composable service/data elements (e.g., dynamic composition mechanisms or discovery based on capability descriptions). Although the ontology supports efficient communication and integration, it does not explicitly describe services or support service/data composition in the excerpt provided. These aspects, particularly service/data description and composition, would require additional semantic modeling to distinguish between service-specific properties and data-specific properties.

### 3.2. Data-Oriented Models

**Figure 12:**
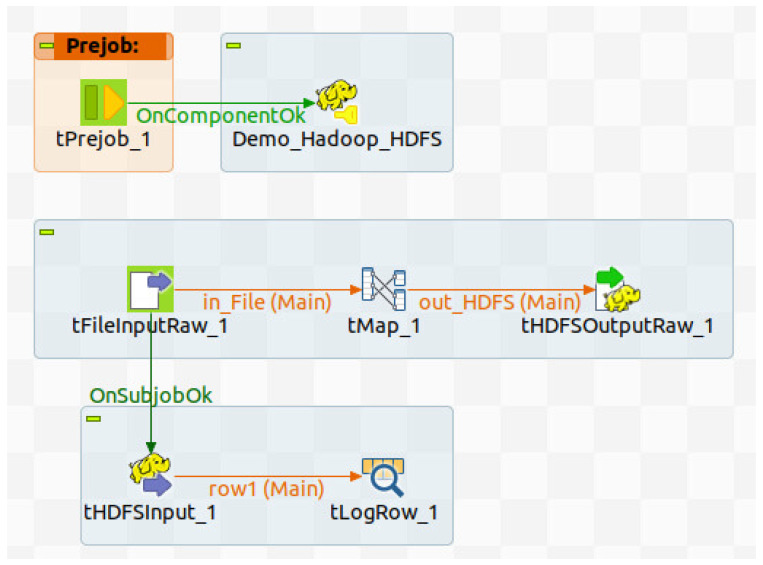
A Talend data composition example for Big Data integration.

Talend (https://www.talend.com/products/data-fabric/, accessed on 28 January 2026) is a unified platform that provides integration, integrity, and governance of data. It has a Talend Trust Score tool that lets users view an assessment of the quality, relevance, and popularity of the data at a glance, allowing them to stay informed and get the data they need. Talend supports connectors for a hundred data sources to collect data, as well as several simple data preparation functions and calculations that can be applied to transform and clean the data. An example of a data composition in Talend for Big Data integration is shown in Figure 12. Talend is mainly used to combine data together, without covering the possibility of composing services together (whenever data is offered by services).

**Figure 13:**
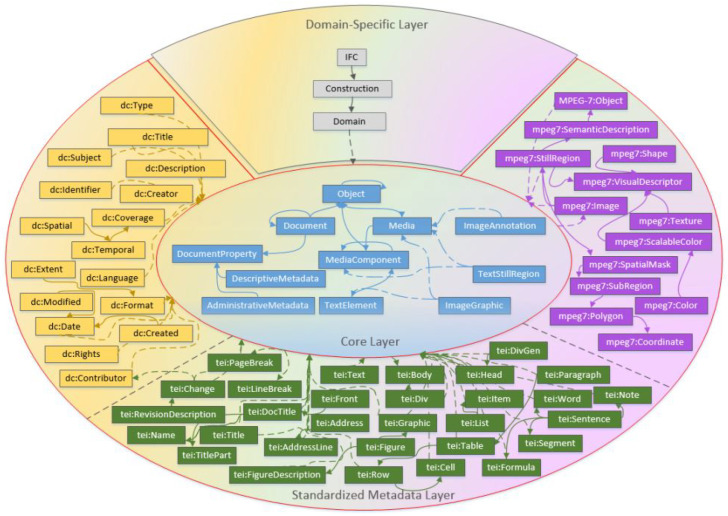
LinkedMDR model representing a corpus of multimedia documents.

LinkedMDR [19] is an ontology model defined for Linked Multimedia Document Representation to describe the knowledge of a heterogeneous document corpus in a semantic data network. The ontology combines existing standards (multimedia-based, image-based, and text-based) used for metadata and content representation, and proposes components to increase their capabilities, forming a model adaptable to different domain-specific knowledge. LinkedMDR, shown in Figure 13, focuses on describing document corpus by using a specific set of low-level entities and relations, without considering data quality, or covering service description (whenever the document corpus is exposed by services). It can be extended to a higher-level data representation, allowing for data composition or/and data-service composition.

The Dublin Core Metadata Initiative (DCMI) (https://www.dublincore.org/, accessed on 28 January 2026) is a key framework for metadata standards, designed to describe a wide range of resources, including documents and images. It offers a simple yet powerful set of 15 core elements, such as Title, Creator, and Subject, aimed at enhancing resource discovery and metadata interoperability. DCMI is extensively implemented in content management systems and the Semantic Web. Its adaptability allows it to be encoded in formats like XML and RDF, facilitating efficient data sharing and integration across platforms. The initiative also supports qualified metadata to provide more precise descriptions, addressing complex needs across various domains. As DCMI continues to evolve, it remains a vital standard in linked data and emerging Web technologies. It can be used in IoT environments, though it may require some adaptation. Its simplicity and broad applicability make it suitable for describing IoT resources, such as devices, sensors, and the data they generate. By using Dublin Core, we can standardize metadata for IoT components, enabling better organization, discovery, and interoperability of IoT data across different systems and platforms. However, for more complex IoT use cases, Dublin Core might be extended by more specialized ontologies (e.g., SSN/SOSA for sensor data) to capture specific details related to IoT.

**Figure 14:**
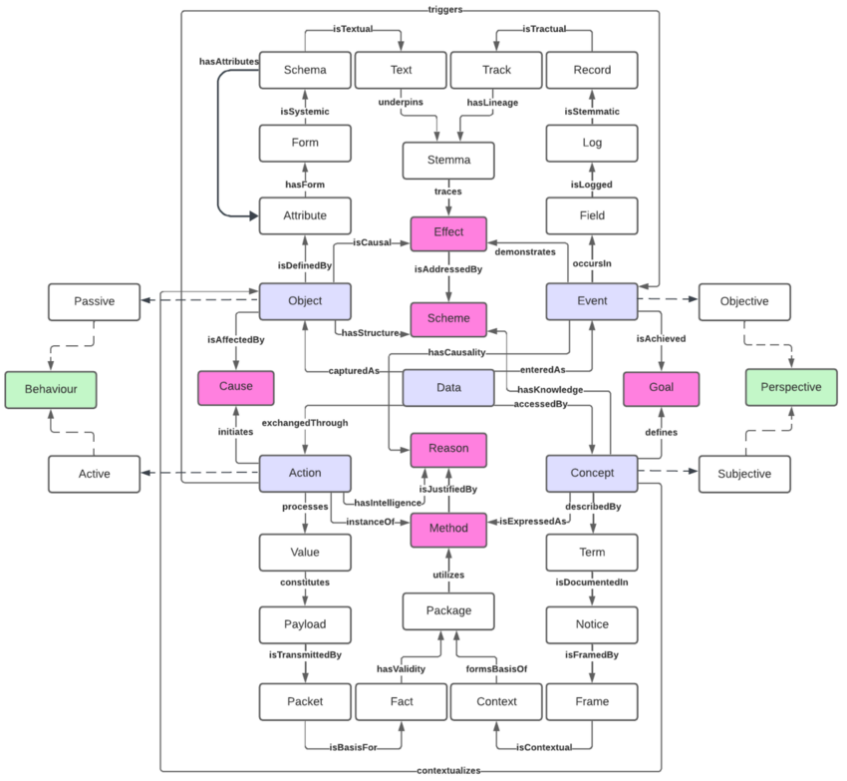
Core Data Ontology.

The work in [20] presents a data-centric framework through an Informatics Domain Model and a Core Data Ontology (CDO) that enhances semantic interoperability, traceability, and knowledge representation in computational systems. The model organizes data into four foundational modalities: Objects, Events, Concepts, and Actions, each capturing a distinct aspect of information. These modalities are formally linked by well-defined semantic patterns, enabling systems to describe and reason about dynamic interactions, intentions, and data transformations. The ontology (see Figure 14), developed in OWL and validated through competency questions, emphasizes intrinsic semantics and role-based access rather than infrastructural or implementation-level concerns. The framework is applicable across diverse domains, including AI auditing, robotics, and enterprise informatics, and is designed to support decentralized, peer-to-peer ecosystems, with potential for domain-specific extensions in areas such as healthcare and the Internet of Things. While the framework provides semantically rich data modeling capabilities, it does not explicitly support the concept of data composition or the linking of multiple data entities in structured workflows. Moreover, it lacks support for describing services as data providers, including properties such as service functions, inputs/outputs, or operational metadata.

**Figure 15:**
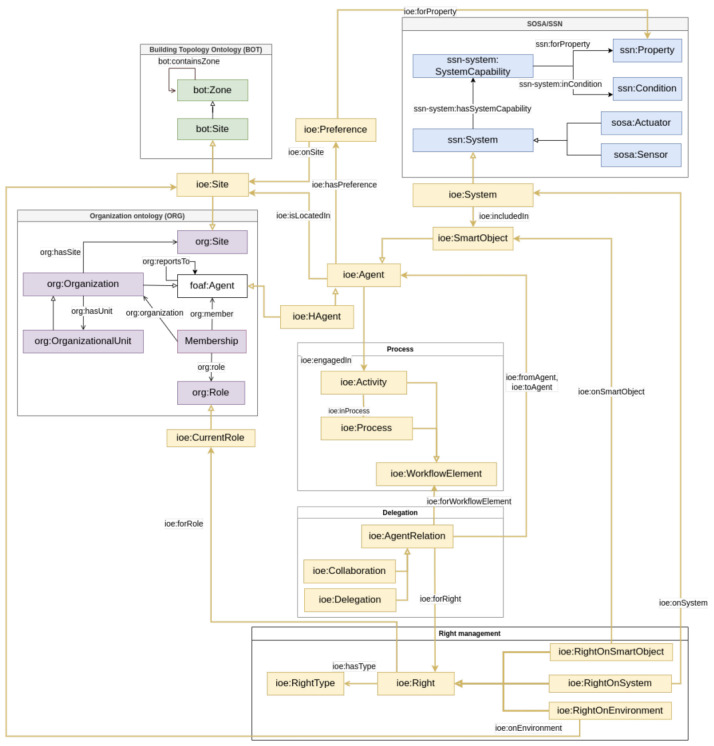
Overview of the SemIoE ontology.

Authors in [21] propose SemIoE, an extensible ontology designed to model Internet of Everything (IoE) environments in Industry 5.0 settings. Built according to reuse and abstraction principles, SemIoE integrates and extends established ontologies, such as SOSA/SSN for sensors, Building Topology Ontology (BOT) [28], the building topology ontology for spatial topology, and ORG (Organization) for organizational structures (https://www.w3.org/TR/vocab-org/, accessed on 28 January 2026), to represent core entities including human and robotic agents, workflows, environmental components, and roles. The ontology underpins an IoE Knowledge Graph and supports several built-in services (e.g., access control, collaboration, secure delegation, environment adaptation) to orchestrate agent interactions (see Figure 15). Although SemIoE provides a strong foundation for modeling entities and interactions, it does not handle the composition of data entities or semantic linking across workflows, nor does it assign service-level properties such as input/output specifications, or metadata detailing operational behaviors within the ontology model.

### 3.3. Comparative Study

In Table 1, we present a comparative analysis of the previously discussed models, based on the criteria outlined at the beginning of Section 3. The symbol “+” indicates positive coverage of a criterion, while “-” denotes a lack of coverage. Firstly, as illustrated in Table 1, none of the existing models fully cover the full set of criteria. Most of these models are service-oriented, focusing on the specifications of elementary services provided by devices (objects). Only a few models address composed services, and these are SOAP-based rather than REST-based. Beyond considering objects that provide services rather than data, most models do not cover services or data exposed by applications. Additionally, none of the models consider virtual resources (services or data) that emulate real-world behavior, nor do they categorize resources to improve environmental exploration and resource management.

Secondly, no existing model adequately covers the expressiveness criteria for both types of resources: services and data. The models are predominantly either service-based or data-based. As a result, all service-oriented models specify the functions provided by the services in their descriptions, and some also describe the services’ Input/Output parameters and the location of the service provider. However, they all fail to define service links, a crucial criterion that can (1) facilitate service discovery and selection, and (2) simplify service replacement when a service becomes unavailable. The only exception is the WoT TD model [14], which supports service links to indicate the next services that can be called in a current service state. However, these links are not semantically defined. For data-based models, some support data linking, while others do not. Similarly, some represent data content, whereas others lack this capability. However, none of these models account for the services or specifications of their data sources. Additionally, existing data links are not sufficiently well defined to enable the generation of new value-added data through integration or composition.

Furthermore, although most service-based models address quality of service (QoS), they tend to focus primarily on the physical and network layers, with limited attention to the application layer. Likewise, the majority of data-based models provide limited or no support for data quality aspects.

### 3.4. Synthesis and Gap Analysis

The models discussed in Section 3 each contribute important elements to IoT/WoT interoperability. However, none offer a complete description of services and data that fully meets the criteria outlined in Section 2. Service-oriented ontologies, such as IoT-O, SSN/SOSA, SAREF, and IoT-Lite, primarily focus on device capabilities. They don’t sufficiently represent application-level services or support composed or virtual resources. Similarly, data-centric models, including LinkedMDR, DCMI, and CDO, emphasize metadata and content representation. They fail to describe the services that create, use, or manipulate this data, and they lack ways to express service–data dependencies. Moreover, none of the existing ontologies define semantically meaningful service links (like *SameAs* or *Follows*) or data relationships (such as *isComplementaryTo* or *hasInCommon*) that would enable automated resource composition, substitution, or discovery. When quality descriptors are utilized, they typically pertain exclusively to physical attributes, seldom encompassing network- or application-level qualities. As detailed in Table 1, no existing model provides, within a singular, cohesive framework, (i) a dual modeling approach for services and data, (ii) support for composite and virtual resources, (iii) semantically defined resource links, and (iv) multidimensional quality descriptors. These deficiencies underscore the necessity for a comprehensive and adaptable semantic resource model, one that integrates services and data, facilitates sophisticated interoperability mechanisms, and allows for automated reasoning across diverse Web-connected environments. The subsequent section presents WoR^+^, our proposed Web resource ontology designed to overcome these limitations.

## 4. WoR^+^ Ontology: Modeling Services and Data

In this section, we present an ontology model, named WoR^+^ (Web of Resources), designed to streamline the discovery, selection, and composition of RESTful Web resources (i.e., services and data) exposed by WoT-connected devices and Web applications. WoR^+^ enables the automation of these processes by standardizing the description of Web resources through a vocabulary that is compatible with a wide range of Web-based solutions and platforms.

Rather than detailing the complete class and property hierarchy inline, we introduce WoR^+^ through a set of core design patterns that capture the main modeling principles and extension points of the ontology. These patterns highlight how resources are exposed, semantically enriched, composed into workflows, and selected based on quality and contextual criteria, as illustrated in Table 2. The complete ontology specification, including detailed class definitions, properties, and axioms, is publicly available in OWL format in a GitHub repository at https://tinyurl.com/bunxt7rr, accessed on 28 January 2026, and the key elements are discussed progressively throughout the remainder of this section.

To preserve readability, low-level class hierarchies, property signatures, and formal axioms are not listed exhaustively in the main text; instead, they are summarized using the design patterns above and fully specified in the public repository. For completeness, the full set of domain and range axioms for the main WoR^+^ object properties is provided in Appendix A.1.

The remainder of this section elaborates on these design patterns, illustrating how they are instantiated and combined within the WoR^+^ ontology.

### 4.1. WoR^+^ Ontology Features

The WoR^+^ ontology captures the properties of Web resources and the relationships between these properties, facilitating their discovery and selection. It also outlines the composition characteristics of resources when they are combined to form a composite resource, whether it is data-based, service-based or both. WoR^+^ ontology builds on several existing models:

• **HSSN**: An extension of the SSN ontology, HSSN models hybrid sensor networks, which incorporate both mobile and stationary sensors. These networks integrate scalar and multimedia features, along with infrastructure and devices that serve as platforms for sensor deployment [24].
• **SOSA**: This ontology models the interactions among elements involved in observation, actuation, and sampling processes [23].

**Figure 16:**
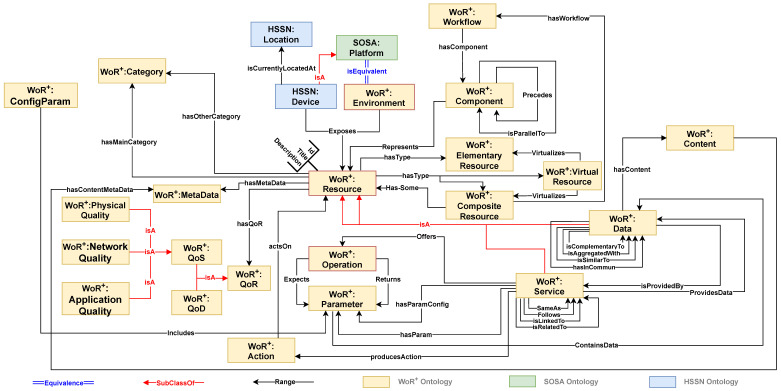
Overview of WoR^+^: The Web Resources Model and its semantic alignment with existing ontologies.

Figure 16 provides a visual representation of how these ontologies are integrated into WoR^+^.

### 4.2. WoR^+^ Ontology Extensions

This subsection details how the core design patterns introduced in Table 2 are realized through concrete ontology extensions. For clarity, these extensions are grouped into three high-level features, each of which implements one or more core design patterns. In particular, WoR^+^ extends the HSSN and SOSA ontologies by introducing additional concepts and relations that realize the Resource Exposure, Semantic Expressiveness, Data Semantics, Location-Aware Resource, and Quality-Aware Selection patterns, as illustrated in Figure 16.

The areas of extension include the following:

(i) **Thoroughness feature** (*Resource Exposure and Workflow Composition Patterns*). This feature focuses on exposing resources (services and data) from devices and application platforms connected to Web environments and assigning categories to these resources. In our model, a resource (*WoR^+^:Resource*) is defined as an RDFS (Resource Description Framework Schema: https://www.w3.org/TR/rdf-schema/, accessed on 28 January 2026) resource type, inspired by the Hydra vocabulary used to describe RESTful services leveraging Linked Data [29]. Each WoR^+^ resource, whether a *Service* or *Data*, can be further classified as an *ElementaryResource*, a *CompositeResource*, or a *VirtualResource*;
(ii) **Expressiveness feature** (*Semantic Expressiveness, Data Semantics, and Location-Aware Resource Patterns*). This feature defines properties related to both service and data resources, including service functions (*Operation*), input and output parameters (*Parameter*), actions (*Action*), and semantic links (e.g., *SameAs*, *Follows*). It also encompasses data content, semantic data relations (e.g., *isAggregatedWith*, *hasInCommon*), and resource locations when exposed by connected devices, which are modeled using *HSSN:Location*;
(iii) **Resource quality feature** (*Quality-Aware Selection Pattern*). This feature captures resource quality through the *WoR^+^:QoR* entity, covering both service-related quality (*QoS*) and data-related quality (*QoD*). Service quality includes physical, network, and application aspects, while data quality addresses factors such as accuracy and timeliness, ensuring that selected resources are reliable and fit for their intended purpose.

#### 4.2.1. Extensions Related to the Thoroughness of the Resource Model

These extensions realize the *Resource Exposure* and *Workflow Composition* design patterns by providing a unified abstraction for exposing services and data from heterogeneous devices and Web platforms, and by enabling their combination into reusable composite resources.

As discussed in Section 3, the majority of IoT-/WoT-based models concentrate primarily on resources that are exclusively exposed by connected devices, largely overlooking those made available by Web applications. To enable the discovery, selection, and composition of various types of resources, we have defined the “Exposes“ relationship in the Web of Resources (WoR+) model. This relationship connects each HSSN:Device and SOSA:Platform entity (the latter equating to *WoR^+^:Environment*) to the *WoR^+^:Resource* concept. A resource, in our work, can be either a Service (*WoR^+^:Service*) or a Data (*WoR^+^:Data*), and has an Id, a Title, and a Description, as related attributes. Furthermore, resources (services and data) can be categorized into different groups, such as “Data Collection Services” for services, and  “Data Collection” for data. Each resource is thus associated with a category (*WoR^+^:Category*), which helps organize the resources and enhances comprehension of their context within the Web environment. In addition, each resource is described by associated metadata (*WoR^+^:Metadata*), which encapsulates supplementary information such as versioning, format, provenance, or licensing. The inclusion of metadata supports advanced resource filtering and improves the precision of resource discovery.

In certain scenarios, a single resource cannot fulfill a user’s request. However, combining multiple resources, known as resource composition, can yield the desired results. To create such a composition, resource discovery and selection must occur prior to a specialized orchestration process that executes the composition [30]. Once the composition is formed, storing it can help avoid rerunning the discovery and selection phases, which are often time-consuming and resource-intensive in terms of CPU and memory usage. As a solution, the Web of Resources model, *WoR^+^*, distinguishes between elementary resources (*WoR^+^:ElementaryResource*) and composite resources (*WoR^+^:CompositeResource*). A composite resource comprises several resources, which can themselves be either elementary or composite. To support the re-execution of stored composite resources, we introduce the *WoR^+^:Workflow* entity, which outlines the execution sequence of resources within a composition. This process includes at least two components (*WoR^+^:Component*) representing the services and/or data involved. The order of these components is defined using the relations “Precedes” and “IsParallelTo”.

To replicate the behavior of real resources that are unavailable or costly to use, we define the *WoR^+^:VirtualResource* entity. It virtualizes both *ElementaryResource* and *CompositeResource* concepts. For example, a virtual service might simulate traffic prediction for testing, while virtual data could consist of synthetic sensor readings when real data is unavailable. These resources support efficient testing and validation in constrained environments. Virtual resources in WoR^+^ are instantiated using synthetic data and simulated service behavior that conform to the same structural, semantic, and contextual constraints as real resources. Synthetic data is generated according to predefined schemas and domain-specific parameters (e.g., data type, value ranges, temporal granularity), ensuring compatibility with the corresponding *WoR^+^:Data* descriptions. Quality attributes associated with virtual data, such as accuracy and timeliness, are derived from controlled generation parameters and explicitly mapped to the *WoR^+^:QoD* metrics. This alignment ensures that virtual resources can be discovered, selected, and composed using the same mechanisms as real resources, while supporting testing, validation, and experimentation in constrained or unavailable environments.

#### 4.2.2. Extensions Concerning the Expressiveness of the Resource Model

These extensions operationalize the *Semantic Expressiveness*, *Data Semantics*, and *Location-Aware Resource* patterns by enriching services and data with functional descriptions, semantic relations, contextual metadata, and spatial information.

The Hydra model served as inspiration for defining a Web resource as an RDFS-typed resource. It is a vocabulary that introduces several concepts for resource description and enables servers to communicate valid state transitions to clients. In this context, Hydra defines the concept of a “Link,” which supports the dynamic discovery of subsequent resources that can be invoked at runtime. However, the model lacks semantic information about the nature of these links (e.g., whether they represent complementary or similar services), which could significantly enhance automatic resource discovery, selection, composition, and substitution when resources become unavailable. To overcome this limitation, the WoR^+^ ontology introduces reflexive semantic relationships between resources. Below are the semantic links that relate services:

- **“SameAs”**: Indicates that the related services offer the exact same functionality.
- **“Follows”**: Signifies that the related services can be executed in a complementary sequence, depending on the functions they provide.
- **“isLinkedTo”**: Denotes that the services can be interconnected based on the compatibility of their input and output parameters.
- **“isRelatedTo”**: Implies that the services are provided by devices located in the same area or zone.

To avoid semantic overlap between service relations, WoR^+^ formally distinguishes the roles of the four links through OWL constraints. *SameAs* captures functional equivalence and is modeled as a symmetric and transitive relation. *Follows* encodes execution ordering and is modeled as transitive, asymmetric, and irreflexive. *isLinkedTo* captures input/output compatibility and is derived through property-chain axioms linking service output parameters to compatible input parameters. Finally, *isRelatedTo* captures spatial affinity and is derived from the co-location of the devices exposing the services. These relations are declared disjoint to ensure that equivalence, ordering, compatibility, and co-location remain formally separable in reasoning and query evaluation. Additionally, the WoR^+^ ontology defines semantic links that relate data elements:

- **“isComplementaryTo”**: Indicates that the data content shares the same subject, which can be specified through associated metadata (*WoR^+^:MetaData*).
- **“isAggregatedWith”**: Signifies that the data is combined with other data to form a unified dataset.
- **“isSimilarTo”**: Denotes content resemblance between data elements (e.g., documents with paraphrased content or images with similar features).
- **“hasInCommon”**: Implies that data shares identical content (e.g., documents with the same paragraphs, or videos with overlapping frames).

##### Domain and Range Axioms

To make the semantics of WoR^+^ explicit and support reasoning-based validation, we define domain and range axioms for the main object properties introduced in the ontology. These signatures serve two complementary purposes: (i) they constrain the admissible usage of relations, preventing unintended modeling patterns, and (ii) they enable OWL 2 DL reasoners to infer implicit types and detect inconsistencies when a property is asserted with incompatible classes.

In particular, the parameter-binding relations *WoR^+^:Expects* and *WoR^+^:Returns* are defined to connect a *WoR^+^:Operation* (domain) to a *WoR^+^:Parameter* (range), ensuring that service interfaces are consistently modeled through input/output parameter specifications. Similarly, service exposure is constrained through *WoR^+^:Offers*, which links a *WoR^+^:Service* to its *WoR^+^:Operation* instances, supporting the explicit representation of functional capabilities. For data resources, *WoR^+^:hasContent* links *WoR^+^:Data* to *WoR^+^:Content*, and *WoR^+^:hasMetaData* links a *WoR^+^:Resource* to *WoR^+^:MetaData*, enabling uniform access to descriptive information independently of whether the resource is a service or a dataset. Finally, workflow control relations are scoped to composition structures: *WoR^+^:Precedes* and *WoR^+^:isParallelTo* are defined between *WoR^+^:Component* instances (domain and range), within a *WoR^+^:Workflow*, ensuring that ordering and parallelism constraints apply only to workflow elements rather than to arbitrary resources. This typing is essential for validating composite resources modeled through *WoR^+^:hasWorkflow* and *WoR^+^:hasComponent*, and for ensuring that each component remains grounded in an actual resource through *WoR^+^:Represents*.

The complete, machine-readable OWL specification (including all property signatures and axioms) is publicly available at https://tinyurl.com/bunxt7rr (accessed on 28 January 2026), and Appendix A.1 provides the full list of domain and range axioms for reproducibility.

##### Formal Semantic Constraints for Key Relations

In addition to property signatures (domain and range), WoR^+^ defines formal semantic constraints for key relations to strengthen reasoning-based validation and prevent unintended modeling patterns. In particular, workflow ordering is constrained through OWL 2 DL property characteristics (e.g., *WoR^+^:Precedes* is modeled as transitive, asymmetric, and irreflexive, while *WoR^+^:isParallelTo* is symmetric and disjoint from *WoR^+^:Precedes*). Category assignment is constrained by enforcing a unique main category per resource (functional *WoR^+^:hasMainCategory*). These constraints are encoded in the OWL specification and validated using OWL 2 DL reasoning. For completeness, a summary of these constraints is provided in Appendix A.2.

The expressiveness model feature also covers several service- and data-related properties. For service resources, WoR^+^ defines the function (*WoR^+^:Operation*) provided by the service (*WoR^+^:Service*). It includes the function’s input parameters (linked via the “Expects” relation between *WoR^+^:Operation* and *WoR^+^:Parameter*) and output parameters (linked via the “Returns” relation). In our model, the *WoR^+^:Operation* entity captures all the necessary details for clients to construct valid HTTP requests when interacting with services. Each operation includes an HTTP method, optional input/output parameters, and descriptive information about the function it provides, such as “Temperature Unit Conversion” or “Energy Consumption Data Prediction”. Furthermore, WoR^+^ introduces the concept of service actions (*WoR^+^:Action*) to explicitly represent the effect a service operation may have on other resources. For example, an action might trigger another service or adjust a system’s state (e.g., adjusting traffic light timing based on real-time congestion predictions). These actions are essential for modeling the dynamic and interactive behavior of services within complex compositions. Moreover, we introduce the concept of *WoR^+^:ConfigParam*, representing the configurable coefficients used to control or adjust the Web services as needed. For example, a configurable coefficient could be the regularization parameter used in optimizing an SVM (Support Vector Machine) service model, which determines the degree of importance assigned to misclassifications. A larger regularization parameter reduces the allowance for wrongly classified examples.

As for data resources (*WoR^+^:Data*), the WoR^+^ ontology defines *WoR^+^:Content*, which refers to the actual information held by the data. This content is central to understanding the nature and purpose of the data, as it conveys the meaning, context, and thematic focus of the resource. For instance, content could correspond to environmental sensor readings, satellite imagery, financial transactions, or textual documents. To further enrich the semantics of data content, the model allows content to be described using metadata elements such as the type of representation (e.g., text, image, audio, tabular) and domain-specific concepts (e.g., “vehicle type” in traffic monitoring or “occupancy level” in smart buildings). These descriptors enable systems to process and reason about data in a domain-aware manner, supporting intelligent functionalities such as recommendation, filtering, transformation, and interoperability across heterogeneous systems.

Furthermore, in addition to the properties defined for each service and data resource, WoR^+^ specifies the resource’s location (*HSSN:Location* concept), which is essential when these resources are exposed by geographically distributed devices. This spatial attribute can influence discovery and selection decisions, particularly in applications like smart cities, disaster response, or environmental monitoring, where proximity and locality matter.

#### 4.2.3. Extensions Concerning the Resource Quality Aspects

These extensions implement the *Quality-Aware Selection* design pattern by modeling service- and data-level quality attributes that support informed comparison and selection of resources.

When multiple resources have some similarities (similar functions in case of services, or similar content in case of data), distinguishing between them to select the most suitable one for a user’s request becomes crucial. To address this, the WoR^+^ ontology incorporates a concept of resource quality (*WoR^+^:QoR*), which can be either services related: *WoR^+^:QoS*, or data related: *WoR^+^:QoD*. In this work, we categorize the *WoR^+^:QoS* attributes into three main groups: (1) Physical Quality (*WoR^+^:PhysicalQuality*), this category describes the attributes of IoT/WoT devices that provide the services, such as battery life and operational range, (2) Network Quality (*WoR^+^:NetworkQuality*), this category covers aspects related to the quality of data transmission between services, including bandwidth and latency, and (3) Application Quality (*WoR^+^:ApplicationQuality*), this category represents the quality of the services delivered, such as response time. Just as it is crucial to define the quality attributes of services, it is equally important to establish the quality attributes associated with data through the concept *WoR^+^:QoD*. Examples of these attributes are: (i) Accuracy, ensuring that data contains correct information; (ii) reliability, confirming that the data originates from trusted sources, particularly if it is sourced from an external database; and (iii) timeliness, verifying that the data is up-to-date, which is vital when working with real-time data.

### 4.3. Workflow-Based Resource Composition

This subsection clarifies how WoR^+^ enables the creation and execution of workflow-based resource compositions, even though Section 4 already explains composite resources and workflows. A composite resource is a structured combination of services and/or data that must be executed, either one after the other or at the same time, to meet complex functional needs. To make this possible, WoR^+^ uses a four-step methodology. (1) Resource Identification. First, relevant resources are found using SPARQL queries. These queries use the semantic descriptions in WoR^+^. These queries help select potential services and data based on their functions, metadata, contextual properties, and QoR/QoD characteristics. This initial phase ensures that the resource pool is suitable for the workflow’s intended function. (2) Compatibility Assessment. Following the selection of potential resources, WoR^+^ assesses their interoperability by examining the semantic *Expects* and *Returns* relations associated with operations, as well as the *isLinkedTo* relation, which indicates compatible input/output parameter connections. This compatibility verification process ensures that the output generated by one resource can be utilized by the subsequent resource, thereby establishing a syntactically and semantically consistent sequence. (3) Workflow Creation. Upon confirming compatibility, the workflow is instantiated as a WoR^+^:Workflow object, which comprises several WoR^+^:Component instances. The execution semantics are represented through relations such as *Precedes*, which denotes sequential ordering, and *IsParallelTo*, which indicates parallel execution. This representation supports both simple and complex compositions, enabling developers to express a wide variety of processing behaviors. (4) Execution and Adaptation. During execution, services may trigger actions (WoR^+^:Action) that update system states or lead to the invocation of additional resources. Throughout the workflow, QoR attributes are monitored to detect performance degradation or failures. In such cases, WoR^+^ supports the substitution of services based on semantic equivalence (*SameAs*) or functional similarity, thus ensuring resilient and adaptive execution. As an illustrative example, constructing a congestion prediction workflow may require integrating vehicle trajectory data, weather information, a predictive analytics service, and a route optimization service. WoR^+^ enables such workflows to be defined, stored, reused, and adapted efficiently, supporting advanced application scenarios across dynamic and heterogeneous Web-connected environments.

## 5. Experimental Evaluation

In this section, we outline the experimental process we used to assess the WoR^+^ ontology. It is founded on 4 parts:

1. *Effectiveness Evaluation:* In which we checked if the concepts and properties established in the ontology could cover the different research goals outlined in Section 5.1 below, and meet the criteria presented in Section 3.
2. *Clarity Evaluation:* In which we aimed to determine whether the names or labels used to describe the concepts and properties are understandable to end users and free of ambiguity (for experts and non-experts).
3. *Performance Evaluation:* In which we evaluated WoR^+^ using quantitative query response time (ms) as the primary metric, measured as the average over 10 sequential runs per query under controlled resource graph configurations (e.g., the increase in the number of devices exposing resources (data and/or services), the increase in the number of resources, etc.). Experiments were executed on Stardog using a Windows 11 machine equipped with a 13th Gen Intel(R) Core(TM) i7-1360P CPU @ 2.20 GHz and 16 GB RAM. The ontology instances and complete executable SPARQL queries used to reproduce the experiments are publicly available at https://tinyurl.com/b59uvjan (accessed on 28 January 2026) and https://tinyurl.com/cksxx5j7 (accessed on 28 January 2026), respectively.
4. *Consistency Evaluation:* This part is used to check if the added concepts/properties generate inconsistencies within the ontology structure (e.g., check if there are concepts that do not have parents).

### 5.1. Effectiveness Evaluation

In this section, we present a set of representative queries (see Table 3) specifically designed to address our research goals across four key dimensions: (1) Exploration, to identify groups of interrelated resources and to enhance understanding of the Web environment; (2) Discovery, to locate resources that fulfill specific user requirements; (3) Selection, to select the most appropriate resources among alternatives that offer equivalent specification (e.g., functionality and content); and (4) Composition/Execution, to integrate the selected resources into a coherent composition and enabling their execution in the correct sequence. Furthermore, we assess these queries based on their effectiveness in meeting the evaluation criteria outlined in Section 3. The queries are expressed in SPARQL (A standard query language and protocol for Linked Open Data on the Web, capable of retrieving and manipulating data stored in Resource Description Framework (RDF) format), as detailed in Appendix A.3.

### 5.2. Clarity Evaluation

**Figure 17:**
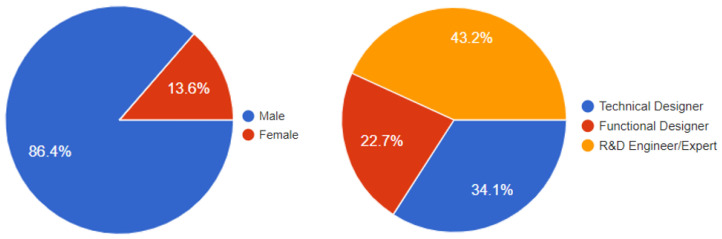
Statistics on participants’ gender and fields of expertise.

To evaluate the ambiguity of the labels used to describe some of the newly defined WoR^+^ concepts and their object properties, we created a questionnaire consisting of 14 multiple-choice questions, in which we proposed several alternative names for the defined service-related concepts and properties, including their compositions (The evaluation of data-related aspects will be addressed in future work.). The questionnaire was completed by 45 male and female participants (i.e., Technical Designers, Functional Designers, and R&D Engineers/Experts). Figure 17 shows the participants’ gender statistics and fields of expertise. Participants reported varying levels of experience in IoT and Web technologies, from early-career practitioners to experts with more than five years of experience, supporting the broader applicability of the findings.

Participants were asked to choose the best name from a list of three synonyms in the domain that best fit each new concept or property, and, if needed, suggest other concepts or properties they found more suitable. The questionnaire was divided into two parts: one part where we proposed several alternative names for some of the WoR^+^ concepts, and another part where we proposed several alternative names for some of the object properties linking concepts together.

**Figure 18:**
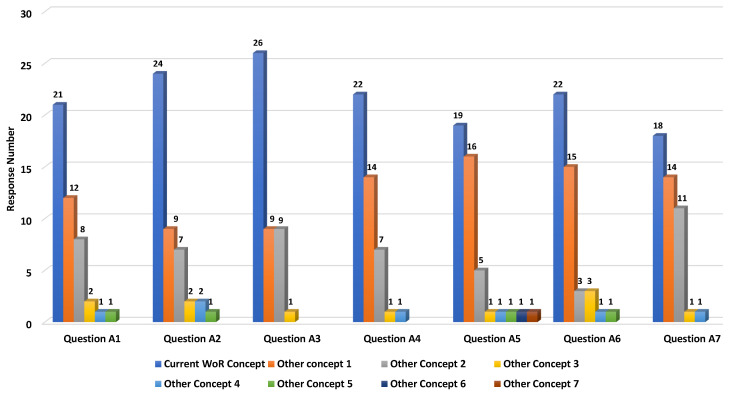
Concepts Evaluation.

Figure 18 shows that the terms that we defined in the first part (for the WoR^+^ concepts) are, in overall, clear for the participants, with 48% selecting the current defined WoR^+^ concepts, 28% choosing the other concepts 1, 16% picking the other concepts 3, and 8% suggesting new terms. The most ambiguous labels were those defined to describe the sequence of services forming a composition (e.g., *WoR^+^:Workflow* in Question A5). As such, the *WoR^+^:Workflow* concept has been selected by 19 participants, compared to 16 selections for the first other concepts presented.

**Figure 19:**
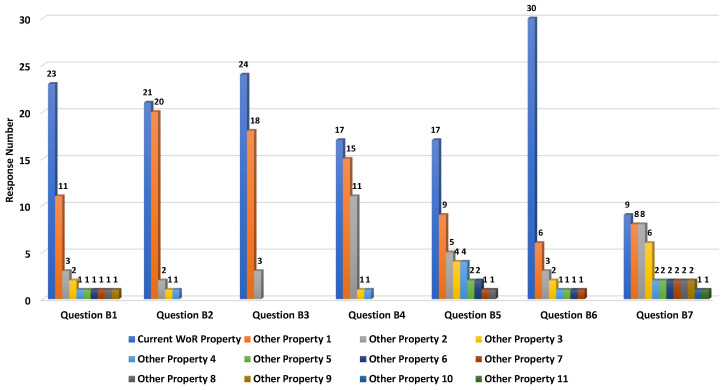
Properties Evaluation.

Figure 19 shows that the labels used in the second part (for the WoR^+^ object properties) were mainly comprehensible for the participants, with 45.5% choosing the current defined WoR^+^ properties names, 27.5% selecting the other properties 1, 11% picking the other properties 2, and 16% suggesting new labels. The most ambiguous labels were those defined to express the relation between the *WoR^+^:Parameter* and *WoR^+^:Operation* concepts to denote the services’ output parameters (Question B2), the relation indicating that two services offer the exact operation (Question B4), and the relation linking two services exposed by devices that are installed within the same location (Question B7). As such, the *WoR^+^:Outputs* property has been selected by 21 participants, compared to 20 members who have chosen the current defined property: *WoR^+^:Returns*. 17 responses have been given to the *WoR^+^:Same-as* property, linking two services that provide the exact operation, compared to 15 responses given to the other property 1: *WoR^+^:Equivalent-to*. As per the property relating to two services exposed by devices having the same location, we obtained 9 answers for *WoR^+^:Is-Associated-to*, 8 answers for both *WoR^+^:Is-Related-to* and *WoR^+^:Is-CoLocated-to* properties, 6 answers for the *WoR^+^:Is-Connected-to* property, and the rest of the responses have suggested new property labels. Due to the small difference between the defined current WoR^+^ property and the rest of the other given ones, we found that it is more comprehensible to use the label *WoR^+^:Is-CoLocated-to*, instead of the current given label *WoR^+^:Is-Related-to*, as it contains a kind of hint about the services’ location device.

The terminology clarity evaluation primarily focused on service-related concepts, reflecting their immediate relevance to discovery and orchestration tasks within WoR^+^. We acknowledge that data-centric concepts (e.g., content, metadata, and data relations) warrant the same level of empirical clarity assessment to fully support the claim of WoR^+^ as a unified model for both services and data. Extending the terminology evaluation to include data-specific concepts is part of our ongoing work and will ensure consistent understandability across both service and data dimensions.

In conclusion, the evaluation shows that the service-related concept and relation labels defined in WoR^+^ are, overall, clear and comprehensible for the 45 participants with different fields of expertise. This clarity supports the reusability and adoption of the WoR^+^ ontology, while ongoing extensions of the evaluation will further strengthen its applicability as a unified semantic model.

### 5.3. Performance Evaluation

This section evaluates how the evolution of the WoR^+^ graph model affects performance in different scenarios. Performance is evaluated based on the execution of a set of queries (selected from those defined in Section 5.1) under various simulated resource graph configurations. This paper focuses on various aspects of data resources and their specifications, as well as on both data and service resources within the model. Scenarios that focus solely on service specifications were addressed in a separate study. Specifically, in this work, we varied: (1) the number of data resources and their associated content metadata (i.e., subject), (2) the number of data and service resources and their distribution in different locations, and (3) the number of data and service resources involved in a composition.

Although baseline comparisons are commonly used in performance studies, defining a fair baseline for WoR^+^ is non-trivial because existing ontologies differ significantly in terms of resource granularity, supported service/data properties, and composition semantics. Many are either service-centric or data-centric and do not jointly model both dimensions with comparable expressiveness. Consequently, we focus on a controlled scalability evaluation using multiple WoR^+^ ontology instances and provide qualitative comparisons against representative ontologies in the related work section.

For each scenario, we report the query response time (in milliseconds) averaged over 10 sequential runs per query. The experiments were conducted using Stardog (https://www.stardog.com/ (accessed on 28 January 2026), an Enterprise Knowledge Graph platform and graph DBMS that supports high availability, high-performance reasoning, and virtualization. All tests were performed on a Windows 11 Home computer equipped with a 13th Gen Intel(R) Core(TM) i7-1360P CPU @ 2.20 GHz processor and 16 GB RAM.

#### 5.3.1. Data and Content Metadata Impact

**Figure 20:**
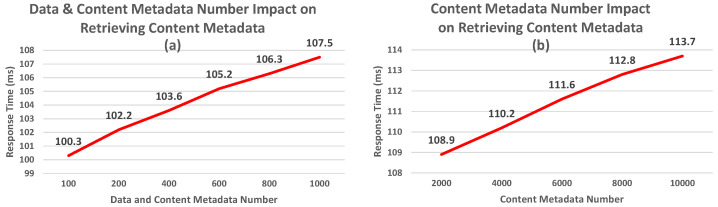
Impact of data and content metadata number on retrieving content metadata.

We examined how varying the number of data resources and their associated content metadata (subjects) affects query performance, as shown in Figure 20. In Figure 20a, we varied both the number of data resources and subjects from 100 to 1000, maintaining a one-to-one mapping between them in each test. In Figure 20b, the number of data resources was fixed at 2000 ms, while the number of content subjects was varied from 2000 ms to 10,000 ms.

For each configuration of the resource graph, we executed Query 2 (see Table 3) to retrieve data resources associated with a specific subject (weather data in our experiments), and measured the query response time.

The results in Figure 20 demonstrate a near-linear increase in query runtime as the number of resources and subjects increases. This growth can be attributed to the added overhead of managing a larger set of searchable items. However, the number of data resources has a more pronounced impact on performance than the number of content subjects. For instance, in Figure 20b, increasing the number of content subjects from 2000 ms to 10,000 ms resulted in a modest 4.8 ms increase in response time. In contrast, in Figure 20a, simultaneously increasing both data resources and subjects from 100 to 1000 led to a more substantial increase of 7.2 ms, indicating that query performance is more sensitive to the number of data resources.

#### 5.3.2. Data and Service Distribution Impact in Different Locations

**Figure 21:**
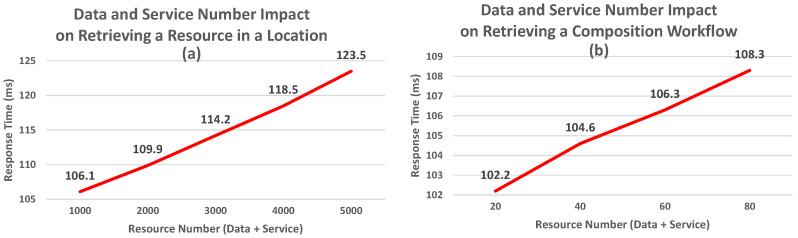
Impact of data and services number on retrieving a resource in a location and a composition workflow.

In the second scenario, we investigated the impact of increasing the number of data and service resources exposed by different devices distributed in various locations (see Figure 21a). In this experiment, we assumed that each device provides either one data resource or one service and is located in a unique, distinct location.

The additional data and service resources, along with their distribution across different locations, clearly contributed to the increase in query runtime observed in the graph. While the growth in response time followed a near-linear trend, the impact of scaling the number of data and service resources across locations is significant. Specifically, the response time increased from 106.1 ms to 123.5 ms (i.e., a difference of 17.4 ms). This effect is more pronounced compared to the previous scenarios shown in Figure 20, where the increase in response time was smaller. In Figure 20a, the difference between the first and last tests was 7.2 ms, and in Figure 20b, it was 4.8 ms. These comparisons emphasize the greater influence of geographically distributed data and service resources on query performance.

To conduct this evaluation, we combined Queries 3 and 11 from Table 3.

#### 5.3.3. Data and Services Impact in a Composition Workflow

In the third case, we examined the impact of adding more resources, both data and services, to a composition workflow (see Figure 21b). In this scenario, we applied Query 17 (see Table 3) to extract the list of data and service resources that together form a composite Web resource, and we measured the corresponding response time. As shown in Figure 21b, the response time increases in a nearly linear fashion as the number of data and service resources grows, reflecting the overhead introduced by retrieving an increasing number of composition components.

**Discussion.** All experiments were conducted using multiple instances of the WoR ontology model, in which we varied the number of resources (data and services) and their associated entities. These ontology model instances are publicly available on GitHub at https://tinyurl.com/b59uvjan (accessed on 28 January 2026). The SPARQL queries used in the experiments are primarily derived from the queries presented in Appendix A.3. For readability, some of these queries are abbreviated or simplified when included in the Appendix A and in the main text. Representative query samples are reported in the manuscript, while the complete, executable versions of all SPARQL queries used in the experiments are publicly available at https://tinyurl.com/cksxx5j7 (accessed on 28 January 2026), enabling full reproducibility of the reported results.

The results from the experimental scenarios demonstrate a consistent and encouraging near-linear relationship between query response time and the various parameters examined. Specifically, as the number of data resources, their associated metadata subjects, and the geographical distribution of data and services increase, the query response time correspondingly rises in a proportionate manner. Similarly, increasing the number of resources involved in composite workflows yields a near-linear growth in response time. This behavior indicates that the WoR^+^ ontology introduces a predictable and controlled performance overhead as the resource graph scales.

From a practical perspective, these response times are well aligned with the requirements of typical Smart Traffic Management Systems (STMS), where semantic queries are commonly executed during resource discovery, contextual filtering, routing decision support, and service orchestration rather than under strict hard real-time constraints. Response times in the range observed in our experiments (tens to low hundreds of milliseconds) are acceptable for operations such as discovering traffic sensors, selecting traffic control services, correlating contextual data (e.g., location, congestion level, or environmental conditions), and constructing composite services for traffic optimization. In such systems, predictable scalability and robustness are often more critical than ultra-low latency, making the observed near-linear behavior particularly suitable.

Beyond scalability, semantic links play a central role in improving the efficiency of resource discovery and composition. Relations such as *SameAs*, *Follows*, and *isLinkedTo* enable early pruning of irrelevant resources and guide composition toward semantically compatible candidates, effectively reducing the search space explored during query execution. In preliminary experiments, queries leveraging semantic links consistently required fewer join operations and evaluated fewer candidate resources compared to link-agnostic queries, indicating improved discovery and composition efficiency. A more extensive quantitative comparison of link-enabled versus link-agnostic workflows is planned as part of future work.

The results also highlight that the most significant impact on performance arises from scaling the total number of distributed resources (both data and services) and their dispersion across multiple locations. This finding is especially relevant for large-scale STMS deployments, where traffic sensors, cameras, actuators, and analytics services are geographically distributed across urban environments. While increases in content metadata and contextual annotations introduce additional overhead, their impact remains comparatively modest, suggesting that semantic enrichment of traffic resources can be achieved without severely degrading query performance.

Regarding limitations, the evaluated scenarios cover resource graph sizes within the bounds explored in this study and rely on simulated and instantiated ontology models rather than live deployments. Nevertheless, this evaluation reflects the typical WoT operational workflow, since resource discovery, selection, and composition are generally performed on semantic descriptions and metadata *before* the actual invocation of services and data retrieval. WoR^+^ is therefore primarily evaluated in its intended role as a pre-execution decision layer for identifying candidates, filtering by context, and constructing semantically compatible workflows. While the experimental evaluation demonstrates the scalability and performance characteristics of WoR^+^ under controlled configurations, real-world operational constraints, such as dynamic resource availability, network variability, and runtime service churn, are not yet captured. Integrating WoR^+^ with real-world Web of Things platforms (e.g., W3C WoT-compliant systems) represents a critical next step toward end-to-end validation in production environments and will help confirm its practical utility beyond simulation-based evaluation. In addition, performance beyond the evaluated scales may be influenced by factors not explicitly studied here, such as significantly larger knowledge graphs, more complex reasoning requirements, higher query concurrency, or deployment in distributed or cloud-based graph infrastructures. At substantially larger scales, techniques such as advanced indexing, query optimization, caching, or graph partitioning may be required to maintain comparable performance levels.

Overall, these observations validate the robustness and scalability of the WoR^+^ ontology and its query mechanisms, supporting their applicability to Smart Traffic Management Systems and other large-scale IoT and Web-of-Things environments.

### 5.4. Consistency Evaluation

Ontology consistency checking is a critical task in ontology construction, alignment, and evolution, as it helps identify and eliminate modeling inconsistencies [31]. To provide a formal evaluation of WoR^+^, we conducted both structural validation and description-logic reasoning checks. First, we adopted SPARQL-based anti-pattern detection queries to identify common inconsistency indicators in the ontology. In particular, Query A detects orphan concepts that have no asserted superclass, while Query B detects abnormal disjointness declarations that may lead to incoherent modeling. The results of these queries revealed no structural inconsistencies in WoR^+^.

Second, we performed OWL 2 DL reasoning using Protégé version 5.6.7 and the HermiT 1.3.8 reasoner to verify ontology consistency and class satisfiability (coherence). The reasoner confirmed that the ontology is logically consistent and that the inferred class hierarchy aligns with the asserted one. In addition, we validated key entailments expected from the model, including the correct classification of resources into *WoR^+^:Service* and *WoR^+^:Data*, and the satisfiability of workflow-related concepts used to represent composite resources. These formal checks provide evidence that WoR^+^ supports sound logical inference and can be safely extended or aligned with domain-specific ontologies in future work.


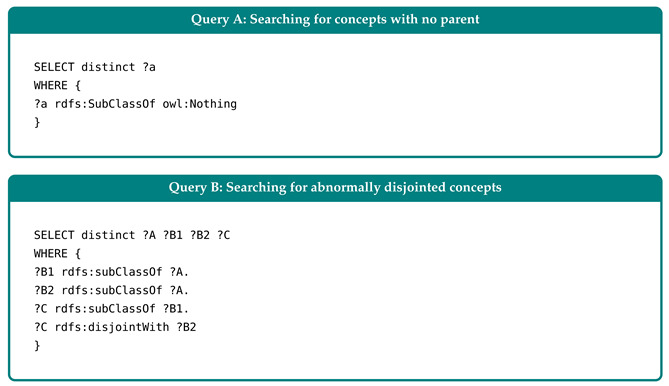


## 6. Conclusions

The Web of Things (WoT) ecosystem, guided by W3C standards, aims to achieve seamless interoperability across diverse IoT (Internet of Things) platforms by enabling device-to-device and application-to-application interactions despite environmental heterogeneity. To effectively identify and utilize the broad spectrum of resources (ranging from data to services) exposed by connected devices and applications, a shared, open, and dynamic knowledge representation is essential. Such a representation must support both syntactic and semantic interactions.

In this paper, we propose WoR^+^, an ontology model designed to describe Web resources, including both Web services and data using a modular and common vocabulary. WoR^+^ facilitates the discovery, selection, and composition of heterogeneous resources offered by connected Web devices and applications. Additionally, it incorporates reasoning capabilities that enable the inference of new knowledge and support extensibility to adapt to evolving domain requirements. We implemented WoR^+^ and evaluated its effectiveness, clarity, consistency, and performance across various resource graph configurations. The experimental results demonstrate promising outcomes, confirming WoR^+^ as a robust framework for managing Web resources in IoT and Web-based environments.

As future work, we will focus on applying and enhancing WoR^+^ in several key areas. First, we plan to integrate WoR^+^ with real-world WoT platforms, such as smart cities, industrial IoT, and healthcare systems, to validate its practical applicability and evaluate its performance in diverse and dynamic environments. Additionally, we aim to incorporate advanced semantic reasoning and AI-driven inference techniques, including machine learning and knowledge graph embeddings, to improve the dynamic, context-aware discovery, selection, and composition of resources. These enhancements will further strengthen WoR^+^ capability to manage complex and evolving resource environments effectively.

## Figures and Tables

**Table 1:** Evaluation of existing IoT/WoT and data-based models with respect to the identified criteria.

	Thorough Model							Expressiveness							Resource Quality	
	Service & Data Oriented	Objects Resources	Applications Resources	Elementary Resources	Composed Resources	Virtual Resources	Categories	Service Function	Service I/O	Service Actions	Service Links	Data Content	Data Links	Resource Location	Service-Based Quality	Data-Based Quality
**IoT-O**	**Service-oriented**	**Limited**	**Limited**	**Limited**	**-**	**-**	-	**+**	**+**	**+**	**-**	-	**-**	**Limited**	**Limited**	-
**oneM2M**	**Service-oriented**	**Limited**	**-**	**Limited**	**-**	**-**	-	**+**	**+**	+	**-**	-	**-**	**-**	**-**	-
**SAREF**	**Service-oriented**	**Limited**	**-**	**Limited**	**-**	**-**	-	**+**	**-**	+	**-**	-	**-**	**-**	**-**	-
**WoT-TD**	**Service-oriented**	**Limited**	**Limited**	**Limited**	**-**	**-**	-	**+**	**-**	+	**+**	-	**-**	**-**	**-**	-
**SSN & SOSA**	**Service-oriented**	**Limited**	**-**	**Limited**	**-**	**-**	-	**+**	**-**	-	**-**	-	**-**	**Limited**	**Limited**	-
**IoT-A**	**Service-oriented**	**Limited**	**-**	**Limited**	**-**	**-**	-	+	-	-	-	-	-	**Limited**	-	-
**Wang Model**	**Service-oriented**	**Limited**	**-**	**Limited**	**Limited**	**-**	-	+	+	+	-	-	-	**Limited**	**Limited**	-
**ForwarDS-IoT**	**Service-oriented**	**Limited**	**-**	**Limited**	**Limited**	**-**	-	+	+	+	-	-	-	**Limited**	**Limited**	-
**IoT-Lite**	**Service-oriented**	**Limited**	**-**	**Limited**	**-**	**-**	-	+	-	-	-	-	-	**Limited**	**Limited**	-
**Semic**	**Service-oriented**	**Limited**	**-**	**Limited**	**-**	**Limited**	-	+	-	-	-	-	-	**Limited**	**-**	-
**Semantic & ontology** **-based framework** **for IoT**	-	**Limited**	-	-	-	-	-	-	-	-	-	-	-	**-**	-	-
**Talend**	**Data-oriented**	**-**	**-**	**Limited**	**-**	**-**	-	-	-	-	-	-	+	**Limited**	**-**	**Limited**
**LinkedMDR**	**Data-oriented**	**-**	**-**	**Limited**	**-**	**-**	-	-	-	-	-	+	+	**Limited**	**-**	**Limited**
**DCMI**	**Data-oriented**	**-**	**-**	**Limited**	**-**	**-**	-	-	-	-	-	-	+	**Limited**	**-**	-
**Core Data Ontology**	**Data-oriented**	-	-	**Limited**	-	-	+	-	-	-	-	+	-	-	-	-
**SemIoE**	**Data-oriented**	**Limited**	**Limited**	**Limited**	-	-	-	-	-	-	-	-	-	**Limited**	-	-

**Table 2:** Core Design Patterns of the WoR^+^ Ontology.

Design Pattern	Purpose	Key Concepts and Relations
**Resource Exposure Pattern**	Provides a unified abstraction for exposing both data and service resources from heterogeneous devices and platforms	*WoR^+^:Resource*, *Service*, *Data*, *ElementaryResource*, *CompositeResource*, *VirtualResource*; relations *Exposes*, *Category*
**Semantic Expressiveness Pattern**	Enables fine-grained discovery, interoperability, and composition by explicitly modeling functional and semantic relationships	*Operation*, *Parameter*, *Action*; relations *Expects*, *Returns*, *SameAs*, *Follows*, *isLinkedTo*, *isRelatedTo*
**Data Semantics Pattern**	Captures the meaning, structure, and relationships of data resources for semantic reasoning and reuse	*Content*, *Metadata*; relations *isComplementaryTo*, *isAggregatedWith*, *isSimilarTo*, *hasInCommon*
**Workflow Composition Pattern**	Supports the construction, storage, and execution of composite resources through explicit workflows	*Workflow*, *Component*; relations *Precedes*, *IsParallelTo*
**Location-Aware Resource Pattern**	Enables discovery and selection based on spatial context in distributed IoT/WoT environments	*HSSN:Location*, *HSSN:Device*, *SOSA:Platform*
**Quality-Aware Selection Pattern**	Supports comparison and selection of similar resources based on service and data quality attributes	*QoR*, *QoS*, *QoD*, *PhysicalQuality*, *NetworkQuality*, *ApplicationQuality*

**Table 3:** A set of useful queries covering the essential goals and criteria.

Query		Goals				Criteria		
		Exploration	Discovery	Selection	Composition/ Execution	Thorough Model	Expressiveness	Resource Quality
**1**	**Retrieve all data resources provided by a Web environment**	**+**	**-**	**-**	**-**	**+**	**-**	**-**
**2**	**Retrieve the data resources whom content is related to a** **defined subject**	**-**	**+**	**+**	**-**	**-**	**+**	**-**
**3**	**Retrieve the data resources exposed in a given location**	**+**	**+**	**+**	**-**	**-**	**+**	**-**
**4**	**Retrieve the data resources that are complementary to other data** **resources**		**+**	**+**	**-**	**-**	**+**	**-**
**5**	**Retrieve all data subjects provided by a Web environment**	**+**	**-**	**-**	**-**	**-**	**+**	**-**
**6**	**Retrieve the data resources that provides content related to a defined** **subject and whose reliability exceeds a value of 75%**		**+**	**+**	**-**	**-**	**+**	**+**
**7**	**Retrieve the services providing a given function**	**-**	**+**	**+**	**-**	**-**	**+**	**-**
**8**	**Retrieve the list of all the services functions provided a Web** **environment**	**+**	**-**	**-**	**-**	**-**	**+**	**-**
**9**	**Retrieve the elementary services provided by a Web environment**	+	-	-	-	+	-	-
**10**	**Retrieve the output parameters of a service and the input parameters** **of another**	-	-	+	-	-	+	-
**11**	**Retrieve the services exposed in a given location**	**+**	**+**	**+**	**-**	**-**	**+**	**-**
**12**	**Retrieve the services that are the same (same-as) as a service**	**-**	**+**	**+**	**-**	**-**	**+**	**-**
**13**	**Retrieve the services that are complementary to (follows) a service**	**-**	**+**	**+**	**-**	**-**	**+**	**-**
**14**	**Retrieve the resources (data and/or services) relative to a given** **category**	+	-	-	-	+	-	-
**15**	**Retrieve the list of all the elementary resource (data and/or services)**	+	-	-	-	+	-	-
**16**	**Retrieve the list of all the composed resource (data and/or services)**	**+**	**-**	**-**	**-**	**+**	**-**	**-**
**17**	**Retrieve the workflow components of** **a composed resource (data and/or service)**	**-**	**-**	**-**	**+**	**+**	**-**	**-**
**18**	**Retrieve the functions provided by the virtual services**	**+**	**-**	**-**	**-**	**+**	**+**	**-**
**19**	**Retrieve the services providing a given function with a** **quality criterion (e.g., Accuracy > 80%)**	**-**	**+**	**+**	**-**	**-**	**+**	**+**
**20**	**Retrieve the services collecting data within a location with** **a quality criterion (e.g., Bandwidth > 400 Mbits/s)**	**-**	**+**	**+**	**-**	**-**	**+**	**+**

## Data Availability

Data are contained within the article.

## Appendix A

### Appendix A.1. Domain and Range Axioms (WoR+ Object Properties)

For completeness and to support reasoning-based validation, we specify the domain and range of WoR^+^ object properties as follows:

- **Exposure and categorization**
  – *WoR^+^:Exposes*: domain {HSSN:Device,SOSA:Platform}; range WoR+:Resource
  – *WoR^+^:hasMainCategory*: domain WoR+:Resource; range WoR+:Category
  – *WoR^+^:hasOtherCategory*: domain WoR+:Resource; range WoR+:Category
- **Service interface and behavior**
  – *WoR^+^:Offers*: domain WoR+:Service; range WoR+:Operation
  – *WoR^+^:Expects*: domain WoR+:Operation; range WoR+:Parameter
  – *WoR^+^:Returns*: domain WoR+:Operation; range WoR+:Parameter
  – *WoR^+^:producesAction*: domain WoR+:Service; range WoR+:Action
- **Semantic links between services**
  – *WoR^+^:SameAs*: domain WoR+:Service; range WoR+:Service
  – *WoR^+^:Follows*: domain WoR+:Service; range WoR+:Service
  – *WoR^+^:isLinkedTo*: domain WoR+:Service; range WoR+:Service
  – *WoR^+^:isRelatedTo*: domain WoR+:Service; range WoR+:Service
- **Data semantics and metadata**
  – *WoR^+^:hasContent*: domain WoR+:Data; range WoR+:Content
  – *WoR^+^:hasMetaData*: domain WoR+:Resource; range WoR+:MetaData
  – *WoR^+^:isComplementaryTo*: domain WoR+:Data; range WoR+:Data
  – *WoR^+^:isAggregatedWith*: domain WoR+:Data; range WoR+:Data
  – *WoR^+^:isSimilarTo*: domain WoR+:Data; range WoR+:Data
  – *WoR^+^:hasInCommon*: domain WoR+:Data; range WoR+:Data
- **Workflow-based composition**
  – *WoR^+^:hasWorkflow*: domain WoR+:CompositeResource; range WoR+:Workflow
  – *WoR^+^:hasComponent*: domain WoR+:Workflow; range WoR+:Component
  – *WoR^+^:Represents*: domain WoR+:Component; range WoR+:Resource
  – *WoR^+^:Precedes*: domain WoR+:Component; range WoR+:Component
  – *WoR^+^:isParallelTo*: domain WoR+:Component; range WoR+:Component
- **Quality modeling**
  – *WoR^+^:hasQoR*: domain WoR+:Resource; range WoR+:QoR
  – *WoR^+^:Includes*: domain WoR+:QoR; range {WoR+:QoS,WoR+:QoD}

The complete, machine-readable OWL specification (including property signatures and axioms) is publicly available at https://tinyurl.com/bunxt7rr (accessed on 28 January 2026).

### Appendix A.2. Formal Semantic Constraints for Key Relations

To improve logical rigor and support reasoning-based validation, WoR^+^ specifies additional semantic constraints for key relations. These constraints prevent unintended modeling patterns and strengthen consistency checking.

#### Appendix A.2.1. Category Assignment Constraints

- **Unique main category per resource:** each *WoR^+^:Resource* has at most one main category
- **At least one main category:** each *WoR^+^:Resource* must have a main category

#### Appendix A.2.2. Workflow Composition Constraints

- **Transitive precedence:** precedence propagates along workflow chains
- **Parallelism semantics:***WoR^+^:isParallelTo* is symmetric and disjoint from *WoR^+^:Precedes*

#### Appendix A.2.3. Service Interface Constraints

- **Operation inputs:** each *WoR^+^:Operation* can expect no *WoR^+^:Parameter*
- **Service interface:** each *WoR^+^:Service* offers at least one *WoR^+^:Operation*

#### Appendix A.2.4. Data Semantics Constraints

- **Content association:** each *WoR^+^:Data* has at least one *WoR^+^:Content* through *WoR^+^:hasContent*
- **Similarity relation:***WoR^+^:isSimilarTo* is symmetric (if a dataset is similar to another one, the reverse similarity also holds)
- **Complementarity relation:***WoR^+^:isComplementaryTo* is symmetric (complementarity is mutual)
- **Aggregation relation:***WoR^+^:isAggregatedWith* is symmetric (aggregation is modeled as an undirected association)

#### Appendix A.2.5. Semantic Links Between Services

- **Equivalence:***WoR^+^:SameAs* is symmetric and transitive

### Appendix A.3. SPARQL Queries Appendix

This appendix provides SPARQL queries that are specifically crafted to support our research objectives across four main dimensions: (1) Exploration, aimed at identifying clusters of related resources to improve comprehension of the Web environment; (2) Discovery, focused on finding resources that meet particular user needs; (3) Selection, which involves choosing the most suitable resources from alternatives providing similar specifications; and (4) Composition/Execution, dedicated to assembling the selected resources into a coherent workflow and ensuring their proper execution in sequence.
